# The Versatility and Diagnostic Potential of VOC Profiling for Noninfectious Diseases

**DOI:** 10.34133/bmef.0002

**Published:** 2023-01-10

**Authors:** Micah Oxner, Allyson Trang, Jhalak Mehta, Christopher Forsyth, Barbara Swanson, Ali Keshavarzian, Abhinav Bhushan

**Affiliations:** ^1^ Department of Biomedical Engineering, Illinois Institute of Technology, Chicago, IL 60616, USA.; ^2^ Department of Internal Medicine, Division of Digestive Diseases and Nutrition, Section of Gastroenterology, Rush Medical College, Chicago, IL 60612, USA.; ^3^ Department of Adult Health and Gerontological Nursing, Rush University College of Nursing, Chicago, IL 60612, USA.

## Abstract

A variety of volatile organic compounds (VOCs) are produced and emitted by the human body every day. The identity and concentration of these VOCs reflect an individual’s metabolic condition. Information regarding the production and origin of VOCs, however, has yet to be congruent among the scientific community. This review article focuses on the recent investigations of the source and detection of biological VOCs as a potential for noninvasive discrimination between healthy and diseased individuals. Analyzing the changes in the components of VOC profiles could provide information regarding the molecular mechanisms behind disease as well as presenting new approaches for personalized screening and diagnosis. VOC research has prioritized the study of cancer, resulting in many research articles and reviews being written on the topic. This review summarizes the information gained about VOC cancer studies over the past 10 years and looks at how this knowledge correlates with and can be expanded to new and upcoming fields of VOC research, including neurodegenerative and other noninfectious diseases. Recent advances in analytical techniques have allowed for the analysis of VOCs measured in breath, urine, blood, feces, and skin. New diagnostic approaches founded on sensor-based techniques allow for cheaper and quicker results, and we compare their diagnostic dependability with gas chromatography- and mass spectrometry-based techniques. The future of VOC analysis as a clinical practice and the challenges associated with this transition are also discussed and future research priorities are summarized.

## Introduction

Olfaction is the oldest human sense, and since ancient times, physicians, such as Hippocrates, Galenus, and Avicenna, have used odor as a diagnostic tool. While the olfactory sense is not customarily used as a modern diagnostic tool, the concept of a characteristic disease odor has reemerged as several studies have shown that dogs can be trained to discriminate lung cancer (LC), breast cancer (BC), prostate cancer (PC), and colorectal cancer (CRC) patients from healthy controls, as well as to discriminate the odor of epileptic seizures from body odors of the same individuals outside of a seizure [1–4]. Olfaction is a complicated process originating from our olfactory sensory neurons in the nose that signal to the brain to identify a smell. Each olfactory sensory neuron has a single odor receptor, and specific microscopic molecules activate each receptor. The majority of these microscopic molecules are volatile organic compounds (VOCs), organic chemicals that have a high vapor pressure at room temperature and consequently evaporate readily under ambient conditions. While VOCs are often associated with characteristic odors, some volatiles are also odorless. VOCs are the end products of cell metabolism and, as such, studying the composition of this volatile fraction of the metabolome, or volatilome, can inform intracellular metabolic status.

Metabolomics is a postgenomic research field that has emerged as a promising approach for the discovery of new disease biomarkers as it is a high-throughput method for analyzing low-molecular-weight compounds in biological samples. Among the different metabolites investigated in recent years, VOCs have emerged as the focus since they are generated within the body and reflect its metabolic processes. Due to their low solubility in the blood, VOCs are transported from the cells in which they were produced through the bloodstream where they can be eliminated in various biofluids including breath, skin secretions, stool, and urine. Exhaled breath, in particular, has been a major biofluid for the analysis of VOCs since it is an inexhaustible waste product that is continuously produced in large quantities resulting in no limits to sample size or frequency. However, exhaled breath is a complicated biofluid, possessing VOCs that can be both endogenous and exogenous. Endogenous VOCs are those produced by the metabolic processes that take place in the body while exogenous VOCs are largely environmental pollutants that have either been inhaled or absorbed through the skin or food ingestion. To overcome these confounding factors in exhaled breath, the gaseous headspace of other biofluids, such as urine, blood, and sweat, were analyzed as well as the headspace of cell cultures reflecting healthy and diseased cells.

Many diseases have been associated with dysregulation of metabolic pathways, and the resulting changes in measured VOC concentrations reflect these metabolic changes. As easily accessible biomarkers are essential for developing a personalized medicine approach for assessment, prevention, and treatment of disease, research into VOCs as noninvasive and sensitive biomarkers for clinical use has been gaining attention. Of particular interest are VOC biomarkers for cancer because cancer-associated gene and protein changes have previously been demonstrated and these changes have a direct effect on the metabolome. As cancer VOC studies have progressed, sample collection methods and VOC analysis instrumentation have evolved accordingly. This review discusses leading sample collection methods and analysis instrumentation as well as summarizes information gained from recent VOC LC, BC, CRC, and PC studies, including reported identities of potential VOC biomarkers. More importantly, it summarizes investigations into the possible underlying biochemical mechanisms that could result in the production of these reported VOCs. Following the discussion of VOC cancer studies, recent VOC studies into other noninfectious diseases, including neurodegenerative diseases, will be discussed. The challenges of identifying possible biochemical pathways involved in the production of VOCs, and the possible solutions identified in the discussed cancer VOC studies, will be discussed as well as other obstacles that must be overcome before VOC biomarkers can gain clinical use.

## Study Design

An extensive literature search was conducted by 3 reviewers using the following criteria: published between 2011 and 2021 (literature search was conducted between August and November 2021); keywords: “VOCs”, “disease VOCs”, “cancer VOCs”, “lung cancer VOCs”, “breast cancer VOCs”, “prostate cancer VOCs”, “colorectal VOCs”, “neurodegenerative VOCs”, “epilepsy VOCs”, “autism VOCs”, and “arthritis VOCs”; databases used: Web of Science and PubMed. Articles were chosen based on their focus on the relationship between VOCs and disease, excluding articles that specifically investigated the effects of environmental pollutants on volatile profiles and markers.

## Sample Collection

**Fig. 1.**
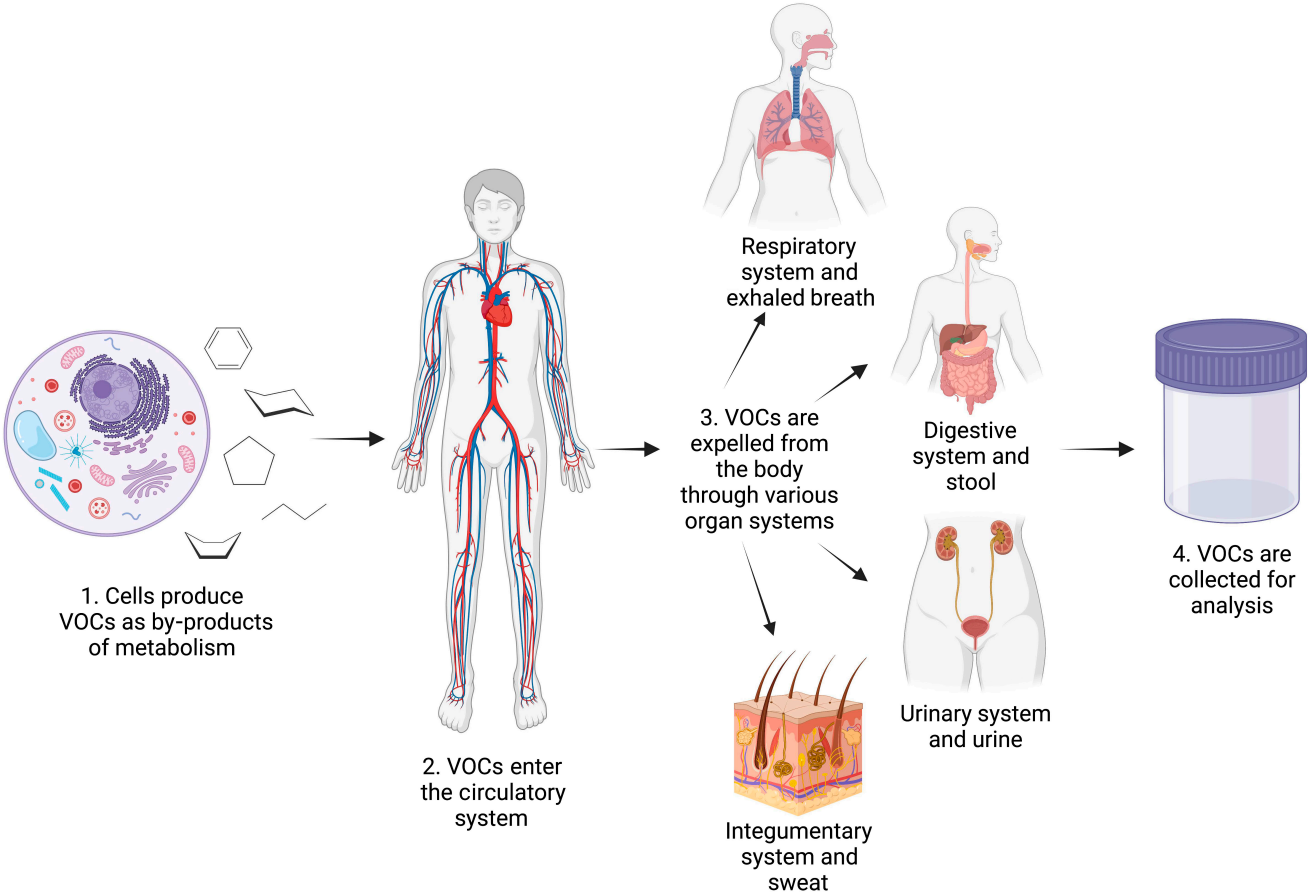
Flowchart showing the various methods of expulsion of VOCs from the body. Created with BioRender.com.

Changes in VOC profiles can be due to various changes in the body caused by diseases. Figure 1 displays the mechanism for the creation of a VOC profile. Alterations in a cell’s structure or function can change the VOCs that are produced and subsequently eliminated from the body through various pathways. The most common pathways are through epithelial skin tissue, the lungs, the gut, and the kidneys; thus, breath, urine, blood, and stool can be collected and analyzed to build a VOC profile. Alternatively, in vitro experiments can identify VOC profiles by sampling cell culture headspace. The various methods of VOC sample collection are discussed in the following section.

### Breath

Breath is the most common biofluid used for VOC analysis. Researchers collect breath samples from patients using a variation of methods based on a face mask or mouthpiece and a bag system. The bag system contains valves and tubes that separate the alveolar air, which is the last portion of the exhaled breath. The system is designed to remove the dead space and environmental air to isolate the alveolar air [5–10]. In many studies, the bag system has been adjusted to fit the study purpose; however, the most common bag used is the Tedlar sampling bag [11–15]. Some example adjustments are creating bags from charcoal or clearing the bags with nitrogen gas before use [9,16]. After sample collection, the VOCs were analyzed utilizing various methods. In several studies, the breath sample was analyzed with variations of the gas chromatography–mass spectrometry (GC-MS) method [16–19], while in others, the breath sample was analyzed with a sensor system, such as an e-Nose [20,21]. Further details about GC-MS and sensor analysis of collected VOC samples are provided in the “VOC detection and instruments” section. In studies using animal models of Alzheimer’s disease (AD), breath was collected by placing sensors next to the animal's mouth [22].

An advantage of analyzing breath samples is that the collection methods are noninvasive, relatively inexpensive, and simple for both patients and researchers [5,6]. The patient simply breathes normally into the system and the resulting air is analyzed [6]. There are many commercially available [19] breath sampling systems, and some of them, such as the ReCIVA Breath Sampler, also help to control environmental contamination and background VOCs [13,17,19]. To maintain VOC accuracy and subtract any background VOCs measured, many studies sampled a controlled reading of the environmental or ambient air while collecting the patient’s breath [20–22]. Although it may be difficult for systems to collect the environmental air at the same time as the patient is breathing, many breath collection systems, such as the ReCIVA Breath Sampler, can simultaneously collect the patient’s breath and the environmental air, which increases the accuracy of the VOCs measured [13,17,19].

A disadvantage of breath sample collection is the accuracy of the VOCs collected from the device due to external patient factors and storage conditions. The accuracy of the sample collected depends on the food or drinks consumed by the patient before taking the sample and the position the patient is in while taking the sample [10,14,18]. In many studies, the patients fasted for between 30 min and 12 h before sample collection to ensure that the VOCs collected were solely from their cells and not from consumed food and drinks [10,13,14,18,19]. Some studies collected breath while patients were sitting at rest to avoid potential alterations in the VOC profile [12,14]. Another disadvantage of breath collection is that the samples should be analyzed as soon as possible after collection because the VOCs quickly deteriorate [10]. A majority of the studies analyzed the VOCs within 24 h of sample collection; however, most studies analyzed the samples immediately.

### Urine

To measure VOC in urine, samples must be collected in a sterile collection container. In studies done on VOCs in PC, CRC, and autism, patient urine samples were collected and frozen at −20°C or −80°C until GC-MS analysis [23–26]. To prepare the urine samples for VOC analysis, the urine was defrosted and, in many studies, combined with an additional solution, such as an acidic or alkaline solution, to alter the sample’s pH level and improve VOC detection and concentration measurement [23,25,26]. In animal studies of BC and LC, the urine was collected by exerting gentle abdominal pressure or collecting fallen urine on parafilm with a pipette [27,28]. Similar to human studies, the animal urine was stored at −20°C or −80°C until GC-MS analysis. However, in an epilepsy study, the animal urine was stored in liquid nitrogen until GC-MS analysis [29].

The advantages of urine samples include noninvasive collection from the patient and ease of sample storage. Urine samples can be stored using various methods without negatively impacting the sample. Although −20°C is the warmest storage temperature reported, there is evidence that urine storage at −20°C has no negative impact on the concentrations of VOCs present in the sample [23].

A disadvantage of urine collection is the need to prepare the sample before VOC analysis. In many studies, an additional solution, such as an acidic or alkaline solution, must be added to the sample to increase the ionic strength of the sample and improve VOC detection [23,25,27]. In addition to adding the solution, multiple tests may need to be run with varying sample pH levels [26]. In studies where multiple analyses were performed on a singlet urine sample with varying pH levels, pH was found to account for the differences in VOC volatility and properties [26].

### Blood

VOCs can be collected from human and animal blood samples obtained through a standard peripheral venipuncture [30–32]. In a study of CRC, blood samples were stored at room temperature and analyzed with GC-MS within 2 h of collection [31]. However, in a study of Parkinson’s disease (PD), the blood samples were stored at 4°C until VOC analysis with GC-MS [32]. Prior to and during VOC analysis with a solid-phase microextraction (SPME) fiber and GC-MS, the samples were heated to 40°C to maximize VOC concentration [30–32]. A benefit of using blood samples is greater control of extraneous sources of variance in the VOC, such as food intake, since the blood can be analyzed before it is affected by external sampling factors [30]. Thus, blood samples obviate the need for fasting. However, a disadvantage of using blood samples is the invasive collection procedure, which may be uncomfortable for some patients, leading to a lower clinical viability.

### Cell culture headspace

**Fig. 2.**
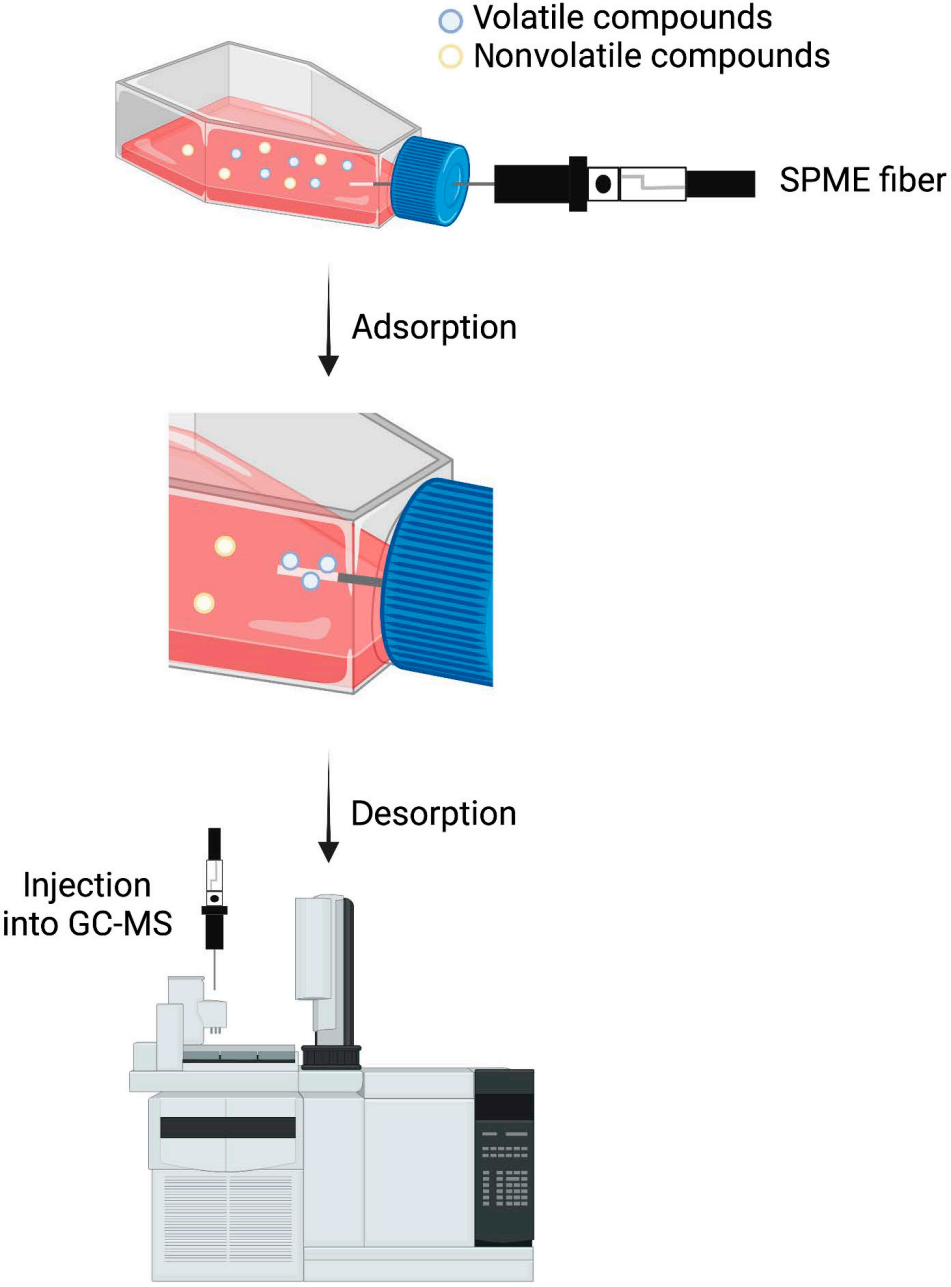
Flowchart showing GC-MS cell culture headspace sampling. Created with BioRender.com.

An alternate method to collecting samples from patients is to collect VOCs from cultured cell lines. To do this, cells are cultured in flasks or dishes until confluency and then, most commonly, analyzed with GC-MS. The cell headspace can be sampled directly from the flask, as shown in Fig. 2, or the cells and the medium can be placed into a headspace vial and analyzed [33–37]. If the cells are used, the sample can be incubated until VOC analysis; however, if the cell’s supernatant is used, the sample must be collected on ice and immediately frozen at −80°C until VOC analysis [34,35]. On the other hand, if the cell headspace is sampled directly from the flask, an SPME fiber or Ultra II SKCTM badge can collect the VOCs from the headspace and they can be analyzed immediately [36,37].

VOC analysis from cell culture headspace studies greatly differ from clinical studies. A benefit of cell culture headspace studies is the elimination of effects from patient factors and the microenvironment. When analyzing the VOCs emitted from a cell’s headspace, the direct measurement from the cells will clearly distinguish if the VOC measurements are associated with the cell and not the microenvironment, indirect metabolic pathways, and patient factors [37]. On the other hand, clinical studies are beneficial for treating patients and determining important genetic information during the treatment plan.

#### Effect of cell culture pH and flask type

When collecting headspace samples from cell cultures, the pH levels and flask type can substantially affect the accuracy of VOCs measured. A study done by Chu et al. [33] investigated the effects of glass flasks and plastic culture flasks on VOC analysis. To conduct the experiment, Chu et al. removed the flask cap, but sealed the flasks with parafilm to prevent the movement of VOCs in and out of the flask. The cell headspace was analyzed with GC-MS, and while 35 distinct VOCs were detected in the empty plastic flasks, only 2 VOCs were detected in the empty glass flasks, which could both be contributed to the parafilm sealing the flasks [33].

pH levels have been shown to influence the efficiency of VOC extraction. Silva et al. [36] cultured BC cells and human mammary epithelial cells in glass flasks and collected the culture medium. The culture medium was adjusted to the following pH levels: 2, 7, or 10 with HCl or NaOH [32], and the VOCs were analyzed from the adjusted samples with GC-MS. The researchers found that the pattern of differentiation at a pH of 10 was similar to the pattern obtained for the flask headspace; however, it was different from the pattern obtained at pH levels of 2 and 7 [36]. These studies show that pH level and flask type can significantly influence the VOCs measured from the samples underscoring the need to report these characteristics in future studies

#### Single-cell headspace

When analyzing cell culture headspace, the measured VOCs are derived from the bulk cell culture. Data suggest that measuring VOCs from a single cell may increase accuracy [34]. In a study done by Serasanambati et al., lung cells were cultured and analyzed as bulk and single cells using GC-MS. The cells were cultured on glass slides and single cells were isolated and placed in headspace vials to be analyzed at various time points. The investigators concluded that single-cell culture sampling was associated with elimination of population heterogeneity, thus isolating potential factors for VOC profiles. Bulk cell culture sampling did not provide sufficient information into the differences in VOC profiles between samples and may have resulted in a wider variation of VOC profiles due to the heterogeneous nature [34]. Therefore, when conducting cell culture headspace sampling, it is important to interpret the findings in the context of the properties of single cell or bulk cell cultures.

### Other methods of sample collection

#### Stool

To analyze VOC samples from stool, researchers collect samples from patients using a stool-specific collection kit. Before providing the samples, patients must not make any dietary changes or take any laxatives. Once the stool sample is collected, it can be stored at −20°C or −80°C until VOC analysis [38–40]. Prior to VOC analysis, the stool sample is thawed and transferred to a headspace vial or bag for VOC sampling [36]. In studies done by Probert and Ratcliffe, Deianova et al., and Batty et al., the stool VOC analysis was done with GC-MS, electronic nose (eNose), and selected ion flow tube mass spectrometry (SIFT-MS) to study nonalcoholic fatty liver disease, the influence of gestational age and mode of delivery on VOC profiles, and CRC [38–40]. A benefit of using stool samples is the ease of sample storage. Research shows that if stool samples are frozen at −20°C within 6 h of stool collection, only minor losses of VOCs occur, which allows for accurate VOC analysis via various methods for several weeks after sample collection [38]. An additional benefit is the noninvasive and relatively efficient collection method.

#### Skin and sweat

VOC samples can be collected from a patient's skin and sweat using multiple methods. The first method is to swab the patient’s body with medical gauze and analyze the VOCs present in the gauze [5,41]. Trivedi et al. and Sinclair et al. utilized this method of sample collection and thermal desorption (TD)-GC-MS to study the VOCs present in PD. To collect the VOC samples, the researchers swabbed the patient’s upper back, sealed the gauze in a background-inert plastic bag, and stored the sample at −80°C until analysis. Patients were asked to not shower 24 h before the sample was collected to ensure accurate collection of VOCs. Prior to VOC analysis, the samples were transferred into headspace vials and incubated to promote the concentration of VOCs present in the sample [41,42].

The second method of collecting VOC samples from a patient’s skin and sweat is to construct a chamber that measures whole-body skin emissions under a controlled environment. This device, constructed by Zou et al. [43], consisted of a test chamber and a transition chamber. Patients sat in the test chamber during VOC collection, and the transition chamber reduced the external environmental impact on the test chamber. The device incorporated various controls, such as background concentration, airtightness, VOC uniformity, temperature, and humidity controls, to ensure that the VOCs collected in the test chamber were accurate. In addition, the device separated the patient’s breath and skin emissions through a respiratory channel and mask that covered the patient’s face and allowed them to breathe into the transition chamber. To collect the VOCs, the patient sat in the device for 30 min and air was pumped through channels into absorption tubes to be analyzed. The air collected in the tubes were analyzed immediately for VOCs with GC-MS. Prior to VOC collection, patients had to follow strict bathing, diet, exercise, and environmental protocols. Three days prior to sample collection, patients could not use any personal care products other than the provided soap, eat anything with an irritating taste, take any medication, drink any alcoholic beverages, exercise vigorously, or be exposed to a highly polluted environment [43].

A benefit of collecting VOC samples from a patient’s skin and sweat is the ease of sample handling. If collecting the sample with a skin swab, the sample can be stored at −80°C until VOC analysis [41,42]. If collecting the sample with the test chamber, the sample is analyzed immediately with the built-in GC-MS [43]. Skin and sweat samples are noninvasive and easy to collect and store.

Some disadvantages of collecting VOC samples from a patient’s skin and sweat are the strict restrictions placed on the patient leading up to the experiment and the accuracy of the sample. Both sampling methods placed restrictions on the patient’s bathing before sampling. However, the device method also restricted the patient’s diet, exercise, and environmental exposure. Some of these restrictions may be difficult and cumbersome for patients to follow, which can result in inaccurate VOC results [43]. In addition, the accuracy of the sample collected can be affected by environmental factors. In the device method, researchers had to conduct multiple tests for each sample to obtain background readings and factor out emissions for other items in the chamber, such as the patient’s clothes and the chair [43].

## VOC Detection and Instruments

For detection of diseases, there are many gold standard screening methods available. However, methods such as computed tomography scanning, mammography, ultrasonography, x-rays, and magnetic resonance imaging (MRI) scanning can be time-consuming, and the accuracy is largely dependent on the physician’s experience, which can lead to misdiagnosis [18]. Recent metabolomics research supports the promise of VOC analysis for disease detection. The methods described below represent analytical, sensor-based, and nanoparticle-based technologies to detect VOCs.

In order to aim this review towards clinical applications, detection techniques included in this review were chosen based on those commonly used in the VOC biomarker studies found in the initial literature search. Based on these findings, papers with detailed explanations of the techniques chosen were found in order to provide a comprehensive summary. As GC-MS and eNose devices were the most used, commercially available services and technologies are also discussed in these sections.

### GC- or MS-based techniques

#### GC-MS

**Fig. 3.**
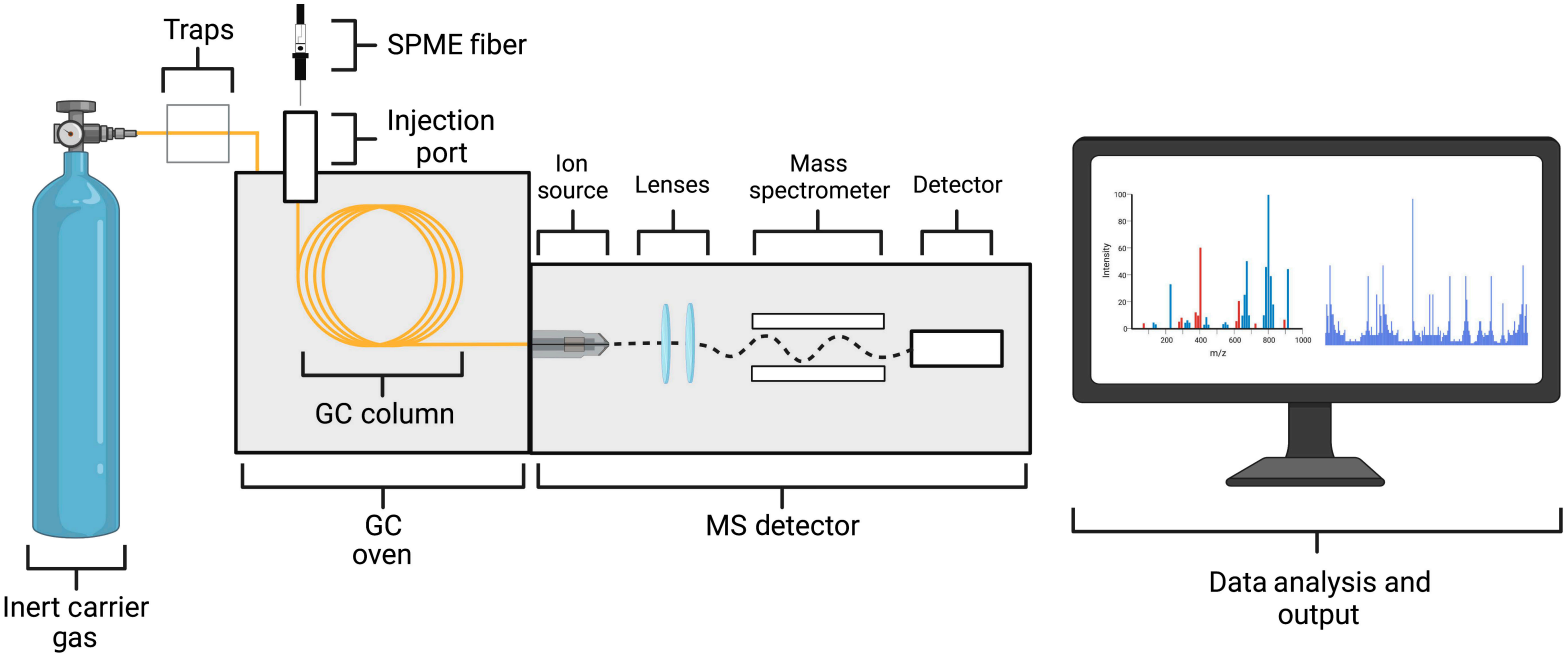
GC-MS schematic diagram. Created with BioRender.com.

GC-MS, a schematic of which is shown in Fig. 3, identifies individual constituent chemical compounds of interest, including VOCs from the collected samples. In GC-MS, VOCs are adsorbed from the sample collection using SPME equipped with a carboxen/polydimethylsiloxane fiber inserted into the gaseous sample for 20 to 60 min [44]. The fiber is transferred to the GC-MS and the VOCs are desorbed (for 2 to 3 min) in the GC injector (200°C). The sample is then separated in the GC column where it will be separated into the individual components. Several parameters such as the split ratio, carrier gas, column temperature and ramp rate, and carrier gas flow rate are optimized for the analysis. The effluent from the column is analyzed in the mass spectrometer, which provides the chemical identity of the compounds by ionizing and breaking the ions into cations. The cations are separated based on different mass-to-charge (*m*/*z*) ratios. By combining the retention index provided by the GC column and the *m*/*z* ratios, the different compounds are matched against a library such as the one provided by the National Institute of Standards Technology (NIST). GC-MS chromatograms are deconvoluted to identify features such as retention time or mass spectra using software such as Automated Mass Spectral Deconvolution Identification System Software (AMDIS) [20,34] or Mass Hunter Unknown Analysis [27,34]. The retention peaks, mass spectra, and Kovats Index [25,35] are compared and matched with the feature information detailed in NIST for the identification of metabolites and spectral library, and VOC scores that meet a score higher than a certain threshold are considered [8,15,26,27,29,31,36]. To confirm that the identity was correct, the nonpolar retention index (NPRI) and NIST index are compared to the experimental NPRI calculated from the average retention time. If the NIST and experimental NPRI values are within 100 units, the compound is deemed identified [27]. If this procedure does not result in identification, the compound is called unknown [15].

GC-MS analysis combined with library search is a powerful technique for compound identification but requires complicated quantitative assessment of the chromatograms. Although the instruments are bulky, expensive, and time-consuming, the major benefits of GC-MS include its high sensitivity level of 100% [25,27,28,35] and its ability to analyze multiple compounds simultaneously to detect chemical differences [45].

Owlstone Medical has developed the Breath Biopsy OMNI as a complete end-to-end service to assist in the discovery and validation of breath VOC biomarkers. OMNI includes the Breath Biopsy Collection Station, as well as support services for study design, data analysis, and data interpretation. The Collection Station contains the ReCIVA Breath Sampler, CASPER Portable Air Supply, and the Breath Biopsy Collect Software. The ReCIVA Breath Sampler works in combination with the CASPER Portable Air Supply to minimize sample variation due to collection procedure and contamination from both the device itself and the environment by providing low background air for inhalation during sampling [46,47]; however, Di Gilio et al. [13] noted in their analysis of LC patients’ breath samples using the ReCIVA that the clean air supply may be a confounding factor due to lung washout. The Breath Biopsy Collect Software controls the pumps within the ReCIVA Breath Sampler according to pressure sensors that learn and respond to the subject’s breathing pattern in order to selectively sample fractions of the exhaled breath that are then absorbed onto an inert-coated stainless-steel tube containing Tenax TA/carbograph 5TD as the sorbent materials [46]. These tubes are stored and sent to the Breath Biopsy Laboratory where the VOCs are recovered using thermal desorption, separated using GC, and analyzed with high-resolution accurate-mass MS. Using custom-made feature extraction and data processing, chromatographic and spectrometric data are reported. Customers also have the option to include univariate or multivariate statistical analysis of VOCs as well as pattern recognition models for the assessment of VOC combinations as composite biomarkers in their report.

#### GC-IMS

The gas chromatograph and ion mobility spectrometer (GC-IMS) includes a GC column, an IMS drift tube, an ionization chamber, and a sensor plate, and the analysis involves 2 stages. The first stage uses GC to separate VOCs based on a chemical interaction with a midpolarity capillary column. The VOCs are detected as they elude from the column using a drift-tube ion mobility spectrometer. The second stage uses IMS to separate ions based on size, mass, and charge after drifting against a buffer gas at a constant flow rate. The output consists of peaks representing retention time as the ions pass through the column and the mobility of the ions as they pass through the drift tube and hit a sensor plate [24]. A needle attached to the GC-IMS allows for vapor aspiration from the sample. The carrier gas flow rate, buffer flow rate, total run time, and temperature of the samples are optimized for the analysis [6,24]. Initially, the carrier gas flow rate is kept lower when the gas sample is introduced and is increased over time.

For point-of-care testing, the GC-IMS technique delivers measurement results in 10 min, collects samples in a patient friendly way, is operable without technical training, and is portable for instant analysis [6,24]. The technique does not require an external gas supply because it is fitted with a circulator gas flow unit. The technique isolates and identifies compounds within the sample, allowing a narrow range of chemicals in the VOC profile [24]. Compared to the GC-MS, the GC-IMS takes substantially less time, is more user friendly, and has a higher potential for point-of-care analysis.

#### SIFT-MS

SIFT-MS is based on spectrometric analysis of VOCs in air or vapor samples by chemical ionization. Precursor ions (H_3_O^+^, NO^+^, and O_2_^+^) are generated in a microwave discharge and an ion is selected by a quadrupole mass spectrometer, which is injected into the helium carrier gas. A sample is introduced into the flow tube, and the precursor ion reacts with the trace gases and the VOCs in the sample. The precursor and product ions are separated, and the count rate of the ions is measured in a detector to achieve real-time quantification [12,39]. The concentration of the volatile compounds is achieved at the parts-per-billion or parts-per-million level by volume [12].

Compared to the quantitative analysis of VOCs of the GC-MS, the benefits of SIFT-MS analysis include a less complicated analysis of VOCs. With the SIFT-MS, data are obtained through scanning a spectrum at a user-defined range of *m*/*z* ratio values and quantification is carried out using a kinetic database stored in the instrument [39]. This combines the product ions derived from the different reagent ions and a tolerance feature that manages ion interferences for every single compound. The analyte concentration is calculated as an average of the lowest product ion concentration and values within a tolerance range. If the product ion falls beyond the tolerance range, then it will not be used in the calculations. The accurate measurements and the VOCs’ relative scales are collected to construct statistical models. The GC-MS approach with the library search is very powerful but involves complex quantitative assessment because it requires precise calibrations of standard compounds if highly specific and reliable quantification is needed. Moreover, the SIFT-MS does not require prior sample collection with an SPME, as does GC-MS, which simplifies the need for sample preparation, preconcentration, and chromatography [12]. Since the SIFT-MS does not utilize the chromatographic separation of the analyte and a library search, the analyzation time is about 30 s per precursor [39] compared to a total run time of 42 min for the GC-MS [23]. The SIFT-MS also does not require any highly technical training compared to the GC-MS. GC-MS is capable of more complex and selective analysis, but SIFT-MS is capable of fast real-time analysis.

#### PTR-ToF-MS

Proton-transfer-reaction time-of-flight mass spectrometry (PTR-ToF-MS) is a real-time monitoring technique of human breath VOC profiles [48,49]. A polypropylene box is connected to a PTR-ToF-MS to provide face-to-face gas supply of synthetic air [50]. Sampling occurs using a heated silco-steel transfer line that is connected to a sterile mouthpiece and placed behind a spirometry flow volume sensor. With PTR-ToF-MS, hydronium ions (H_3_O^+^) are produced from pure water vapor as primary ions. After which, a proton transfer reaction occurs in a subsequent drift tube and is transferred from hydronium ions to molecules with relatively higher proton affinity. Ions are focused further by a series of lens systems and then pulsed towards a high-resolution reflectron time-of-flight mass spectrometer to be detected by a microchannel plate detector. Flow rate, time resolution, transfer line, drift tube temperature, drift tube voltage, and drift tube pressure are all optimized for best results.

Compared to a GC-MS, a PTR-ToF-MS allows continuous real-time measurements without a need for sample preparation resulting in a faster analysis time without a trade-off in sensitivity [50]. However, a PTR-ToF-MS cannot be used for the purpose of concentration changes especially for clinical diagnosis for a reliable and accurate quantification. Trefz et al. [48] showed that the PTR-ToF-MS is very sensitive to trace gases in the sample air such as O_2_ or CO_2_. When the amount of oxygen in the experiments increased, the H_3_O^+^ count significantly decreased by more than 40%. Therefore, varying oxygen concentrations introduce differences in the measured signal intensities of the PTR-ToF-MS [48]. Lower levels of oxygen can be neglected; however, a continuous supply of oxygen concentration such as by a ventilator in a clinical setting cannot be neglected. Therefore, while PTR-ToF-MS allows the assessment of VOC profiles and physiological changes, it is not a powerful enough technique to be utilized in clinical settings for an accurate and reliable quantification.

#### FAIMS

Field asymmetric ion mobility spectrometry (FAIMS) involves a gas detection process that separates chemical ions within a mixture of VOCs based on their movement in high electrical fields [24]. The analytical technique takes a physical measurement of the molecules instead of measuring the chemical interaction between the molecules [51]. The FAIMS technology consists of ionization, ion separation based on mobility, detection, and exhaust. After the sample is introduced through a carrier gas, it passes through the ionization region where the components are ionized via a charge transfer process or by direct ionization to form both positive and negative ions. The ions pass through parallel plates and a radiofrequency waveform is applied to create a varying electric field under which the ions follow different trajectories depending on their mobility. A direct current voltage is applied through the electrode channel to shift the trajectories to the path of the detector to allow more ions to reach it. The number of positive and negative ions recognized by the detector is representative of the concentration of the chemical in the sample [24]. The flow rate over the sample, the compensation voltage, and the dispersion field that represents the magnitude of the electric field are optimized for best results. The compensation voltage is set to remove the effect of the drift produced by a high electric field [51].

An advantage of the FAIMS is that it allows gas molecules to be separated and analyzed at atmospheric pressure and room temperature unlike the GC-MS. Compared to the GC-MS, FAIMS can be applied without any technical training, making it a useful tool for point-of-care testing.

### Sensor-based techniques

#### Metal oxide chemiresistive sensors

Target gases are detected using metal semiconductor metal oxide sensors via redox reactions between the gas molecules and the sensing materials. When the sensor is exposed to the air, oxygen gas molecules are absorbed on the surface of the sensing material forming various oxygen species (O^2−^,O^−^,and O_2_^−^). The oxygen species are formed by capturing electrons from the conduction band leading to an electron-depleted region and band bending on the surface of the semiconductor metal oxide. The loss of electrons results in an increase in the resistance of the sensor. When the sensors are exposed to gases, the trapped electrons are released back to the semiconductor metal oxide resulting in a decrease in resistance and increase in the carrier concentration [52]. The morphology of the metal-oxide semiconductor gas sensors directly impacts the sensitivity of the sensors because of the interaction between VOCs and adsorbed oxygen molecules. The performance of the sensors can be enhanced by adjusting the microstructure, defects, catalyst, heterojunction, and humidity of the metal oxide semiconductor gas sensor [52]. Lavra et al. [44] introduced a self-adaptive temperature modulation coupled with a metal oxide semiconductor gas sensor to adjust the working temperature modulation of the sensors according to the gas response, resulting in a stabilized response over an extended period of time and improved sensor sensitivity.

While a GC-MS is able to distinguish between small differences in VOC signatures between molecular expressions, the sensors cannot discriminate between small VOC differences. However, with continued improvements in the sensitivity of the sensors with techniques such as temperature modulation, the sensors are able to capture differences in VOCs with high accuracy [44]. Generally, compared to a GC-MS, the sensors are cheaper, less-time consuming, and portable.

#### Nanoparticle-based sensors

Chemiresistors, based on composites of organically capped or cross-linked gold nanoparticles are used to detect VOCs [53]. In a sensor array of chemiresistors, each sensor shows either a positive or a negative resistance change as a response to various odorants [7]. The design and fabrication of the nanosensor array developed by Peng et al. included a modified 2-phase method with a ligand-exchange procedure. Fourteen cross-reactive nanosensors with different organic functionalities were mounted on a polytetrafluoroethylene circuit board that made up the nanosensor array [7]. For measurements, the developed nanosensor array was mounted on a polytetrafluoroethylene circuit board inside a stainless-steel test chamber with a 100-cm^3^ volume. The test chamber was first evacuated, then a gas sampling system delivered a pulse of breath from the sample bags to the chemiresistive sensors, and then evacuated again. In a typical experiment, signals of the gold nanoparticle sensor array were collected for 5 min in baseline response of vacuum, then for 5 min during headspace sample exposure, and then again for 5 min in vacuum. Each sensor of the array went through a reversible change in electrical resistance when exposed to the sample breath. The response of each sensor was unique because each is composed of different chemical materials. To measure the resistance of all the sensors simultaneously as a function to time, an Agilent multifunction switch 34980 was used [37].

A disadvantage of the nanomaterial-based chemiresistive sensors is that the arrays of broadly cross-reactive sensors are ideally suited to trace the cancer odor prints directly but cannot identify the individual compounds as a GC-MS would. Moreover, some of the nanomaterial-based chemiresistive sensors have a detection limit of 1 to 5 parts per billion (ppb) to typical cancer biomarkers but others may have a detection limit down to 10 parts per trillion [7]. Analysis with the chemiresistive sensor arrays requires no pretreatment of the breath samples such as preconcentration or dehumidification, and the test can be carried out without any technical training. Also, the sensor array is insensitive to factors such as age, gender, lifestyle, nutrition, medication, and smoking habits. It is a cost-effective, easy-to-use, portable, and noninvasive diagnostic tool for detecting cancers through a single breath test [7].

In the effort to develop novel sensors for detecting VOCs, metal-organic frameworks (MOFs) have emerged as a potential candidate for fabricating biosensing systems [54]. Some of the physical–chemical properties of MOFs that make them appropriate for applications in chemical sensors are their adjustable pore size, small crystal density, and unique network structures that can form by self-assembly [54,55]. Furthermore, MOFs exhibit fluorescence in response to various small molecules, a property exploited by Xia et al. to implement fluorescent detection of LC VOC biomarkers. A disadvantage to MOFs is their low thermal stability and high moisture susceptibility, and Xia et al. overcame these shortcomings through postsynthetic modification to introduce *N*,*N*′-dimethylethylenediamine and *N*,*N*-dimethylaminoethylamine, 2 amines with the same molecular formula but different structures, into the MOFs. This postsynthetic modification allows for the alteration of the physicochemical properties of the MOF while retaining its crystal structure [54]. As a zeolite imidazole framework (ZIF-8) is one of the most stable MOFs, and as it has been extensively studied, it was the chosen MOF by Xia et al. Chen et al. [55] also chose ZIF-8 as their MOF for studying VOCs and decided to couple Au nanoparticles and ZIF-8 in order to detect VOCs using surface-enhanced Raman spectroscopy. While each study is using a different approach to analyze VOCs using an MOF, both are promising strategies for developing biosensing systems for VOC detection.

A disadvantage to using the amine-functionalized ZIF-8 as a fluorescent probe is the variety of fluorescent sensitivity between prospective LC biomarkers due to the steric hindrance of the introduced functional groups [54]. The functional group used for modification of the MOF must be designed and studied for each disease and may prove to be variable between patients, which would further complicate the fabrication process. Similarly, a disadvantage to using AuZIF-8 nanoparticles is knowing the appropriate shell thickness for effective probing of VOC biomarkers. While Chen et al. concluded that a shell thickness of 3 nm was appropriate for characterizing VOCs, they only reported surface-enhanced Raman spectroscopy spectra for benzene derivatives, a small class of biologically relevant VOCs. Advantages to nanomaterial-based sensors include their small characteristic dimensions (1 to 100 nm) and large surface-to-volume ratios, unusual target binding properties, and structural robustness [10]. Moreover, organic components in the sensors provide sites for the sorption of analyte molecules, which allows a control over the interparticle distance. This allows uniform interparticle distances in the composite films resulting in achieving controlled signal and noise levels [7]. Overall, both procedures would result in low-cost, rapid biosensing platforms; however, the reliability of these platforms for sensing a variety of VOCs is still questionable.

#### Colorimetric sensors

A colorimetric sensor array consists of chemical sensors composed of chromogenic reagents, which cause a change in the sensors’ colors based on the analyte absorptions into the sensor system [56]. Different spots (typically 36) in the sensor array are made of different chemically sensitive compounds. The broad sensitivity system ranges from lower parts per million to upper parts per billion. Conventional colorimetric sensor arrays use a range of chemically responsive dyes such as solvatochromic dye or fluorophore and metal oxides [57]. However, they generally require a high operating temperature and result in low selectivity and stability against changes in humidity. Therefore, newer colorimetric sensors are constructed from diverse chemoresponsive dyes such as Lewis acid–base dyes, Bronsted acidic or basic dyes, dyes with large permanent dipoles, redox-responsive dyes, and chromogenic aggregative colorants [57]. For analysis, the sampled exhaled breath was drawn over the sensor array for ~10 min. The changes in the red, green, and blue spectrum were then converted to numerical values for observation [45,56]

A limitation of the cross-reactive sensor systems is that they identify the entire mixture of breath chemicals and cannot individualize components of the mixture. Unlike techniques such as GC-MS, PTR-ToF-MS, and FAIMS, the colorimetric sensor array does not explain the origin of the chemicals. However, the sensor array can identify a multidimensional discriminatory pattern of breath analytes in an inexpensive and portable way using lower operating temperatures and can provide visual results without any advanced training.

#### SiNW FETs

Silicon nanowire field-effect transistors (SiNW FETs) provide an opportunity to detect chemical and biological species. A SiNW-FET is composed of a single SiNW connected between a source and drain electrodes, a PDMS channel on the device that delivers the sample, and a lock-in amplifier that records the electrical signal [58]. When positively charged targets bind on the sensors, the holes are accumulated in the SiNW, which leads to an increase in the electrical conductance, and the negatively charged targets cause a decrease in the electrical conductance. However, traditional SiNW FETs exhibit detection limits of 20 ppb NO, but the same devices showed no response to 40 ppb hexane (a nonpolar VOC). Therefore, SiNW FETs respond weakly to nonpolar VOCs. Emerging SiNW FETs that are molecularly modified with the appropriate organic receptors have demonstrated their ability to detect and classify several diseases [59,60]. In a study done by Paska et al., SiNW FETs were modified with a silane monolayer by coating with an appropriate hydrophobic organic hexyl trichlorosilane (HTS) to enhance the sensitivity towards nonpolar VOCs [59]. Similarly, in a study done by Shehada et al., SiNW FETs coated with trichloro(phenethyl) silane and heptanoyl chloride could detect VOCs linked with the breath print of the diseases and distinguish between LC and gastric cancer, and between asthma and chronic obstructive pulmonary disease [60]. The SiNWs are prepared by a vapor–liquid–solid growth technique. A stainless-steel test chamber contains the sensor array, and a manual thermal desorption (TD) is used to transfer the VOCs trapped in a disposable sorbent tube at a constant flow rate.

Modified SiNW FETs offer high sensitivity, low-power consumptions, and fast-response times, and are compatible with silicon technology [60]. They are also miniature in size and therefore scalable, making the sensors a functionable and portable technique for the detection of VOCs. While sample results were satisfactory, further clinical trials with an increased sample size using Si NW FET sensors that enable in situ sampling and analysis are required to confirm the breath prints.

#### eNose

eNoses are composed of an array of sensors that imitate mammalian olfaction to obtain a complete spectrum of the VOC profiles sampled from specimens such as breath, blood, urine, and the headspace of cell cultures. The interaction between the sensor array and the VOCs upon odor exposure results in a change of the electrical resistance in each sensor. The change in resistance produces a profile of the gas mixtures known as the smell print. There are different commercially available eNose devices with different sensor clusters designed for various applications; however, the objective of obtaining reproducible measurements of VOC profiles remains the same.

**Fig. 4.**
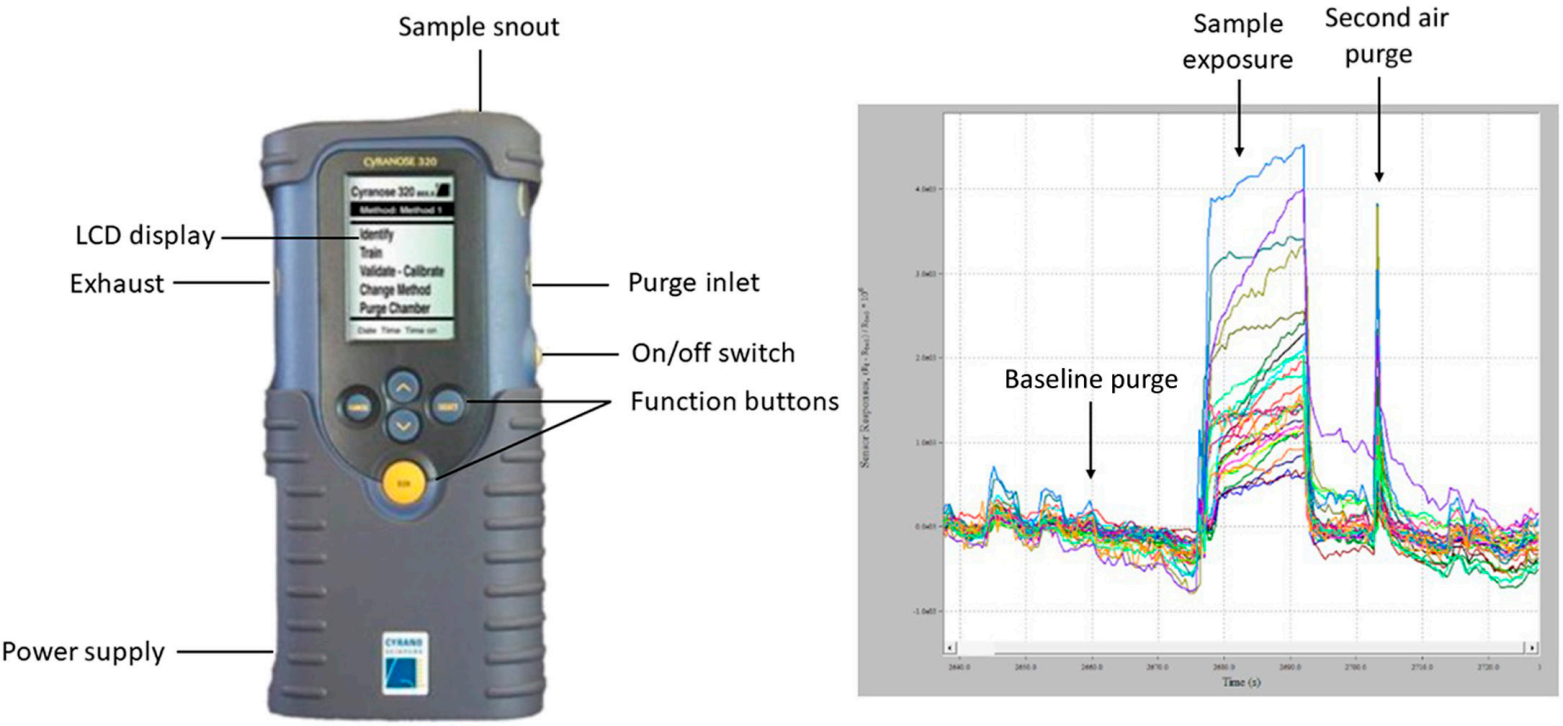
Cyranose 320 device (left) and example output (right).

The commercially available Cyranose 320, shown in Fig. 4, consists of a nano-composite array of 32 organic polymer sensors (Fig. 4). The electrical resistance of the sensor array is altered when the polymers swell after exposure to the VOCs [11]. Each sensor has a unique polymer coating, which results in a competitive interaction between the sensors and the VOCs [40]. The changes in the electrical resistance are dependent on the sensor material and the chemical composition of the VOC. The changes are captured as raw data representing a unique smell print that can be further used for pattern-recognition analysis. The first step with the Cyranose 320 eNose involves purging the sensors with filtered air derived from ambient air passing through a filter to obtain a baseline reference signal. The measurement of the sample involves continuous airflow through an airtight loop system to prevent ambient air dilution. After the sample measurements, the sensors are purged. The time for baseline purge, sampling, air intake purge, sample purge, and pump speed are optimized based on the sample measurements. Usually, the temperature of the sample is kept at 37°C or above. For exhaled breath measurements, a Tedlar bag containing the sample breath can be connected to the eNose for sampling [15,61].

Another eNose technology is the ChemPro100 eNose, which is an ion mobility spectrometer-type flow-through system consisting of 2 sensor clusters producing measurement data in both relative and absolute scales. The sensor clusters include an ion mobility cell that contains 8 electrode strips producing 16-channeled output and a metal oxide-based semiconductor cell that produces 2 channels of output. The device also includes humidity and temperature sensors and a continuous flow pump [62].

The Aconose is an eNose device consisting of 3 micro hotplate metal-oxide sensors and a Tenax tube. When exposed to the breath samples, redox reactions of the VOCs occur on the sensor surface changing the conductivity of the sensor [21]. The measurements follow a temperature cycle profile, and the change in conductivity is recorded within the temperature cycle, and each cycle is repeated multiple times.

A biosensor-based eNose device developed using genetically engineered DNA from nematode worms, the Cybernose sensor uses an array of biological receptors rather than solid-state sensors. The receptors within the Cybernose change shape when an odor molecule binds to them and subsequently emits a mixture of blue and green light that can be measured using optical sensors to indicate the presence and amount of a particular substance in the sample [63].

The LibraNose 2.1 is a quartz microbalance (QMB) 8-sensor array eNose device [64,65]. QMB sensors are used in electronics to drive oscillator circuits. Each QMB sensor is functionalized with a layer of metalloporphyrins that act as receptors for the sensor [5]. When the sensor is exposed to the sample, the molecules bind to receptor sites to change the mass of the sensor. A change of the mass gravitating onto the quartz surface results in a change of the frequency of electrical signal of the oscillator circuit of which the sensor is connected [5].

Compared to analytical technologies such as GC-MS, an eNose device is not capable of accurately identifying and quantifying specific measured VOCs. However, eNose can detect a wider and more complex mix of VOCs as compared to the GC-MS. GC-MS is also expensive and time-consuming and requires technical expertise to operate, which is not ideal for clinical diagnostics. The eNose is faster, cheaper, and portable, making it a suitable device for point-of-care screening for the early detection of diseases.

## VOC Analysis for Disease Diagnosis

Genomics is the study of a person’s genes, also known as the genome, and metabolomics is the study of all the small molecules involved in the metabolic pathways in the body, also known as the metabolome. VOCs are products of the metabolome. While studying the genome has been a major aspect of understanding human biology, genes serve as the blueprint for biological function and many diseases do not alter a person’s genetics. As such, genomics can be used to predict a person’s risk of developing a disease in their lifetime. Metabolomics, on the other hand, can be used to detect disease currently in the body and the stage and presence of a disease in the body. Furthermore, by monitoring the changes in the metabolome through the analysis of the VOCs it produces, the progression of the disease and the person’s response to treatment can be monitored. The ability of metabolomics to provide real-time insight into a person’s metabolic condition has increased interest in metabolic biomarkers, such as VOCs, rather than genetic biomarkers, that only inform susceptibility to disease [66–69]. Biological markers, or biomarkers, serve as indicators of the cellular, biochemical, and/or molecular alterations in the body as well as indicators of normal biological processes, pathogenic processes, and/or pharmacological responses to therapeutic intervention [70]. Practically, biomarkers are used to inform risk assessment, diagnosis, progression, and outcome of disease; however, many biomarkers are measured from human tissue or cerebral spinal fluid, both of which involve invasive procedures to collect. VOCs are a potentially noninvasive biomarker, but the lack of understanding regarding how reported prospective VOC biomarkers are produced by the body undermines their clinical viability. This section of the review focuses on identifying potential VOC biomarkers for disease reported in the last 10 years and investigating the hypothesized biochemical mechanisms that could result in their production.

### Cancer

Cancer is one of the diseases most commonly investigated using VOC techniques. As a result, most research articles and review articles regarding VOCs focus on their application in cancer diagnosis. This section of the review summarizes recently published data regarding LC, BC, CRC, and PC, as these are the 4 most common cancers [71], and assesses the correlations between studies using cell-based, animal-based, and human-based experiments. VOC biomarkers found by multiple research groups under varying experimental conditions are reported, and their possible underlying biochemical pathways are explored.

#### Lung cancer

LC is the most commonly diagnosed cancer worldwide, followed by BC, CRC, and PC [60]. Many VOC studies focusing on identifying LC biomarkers use exhaled breath as their sample source since it can be noninvasively collected and is directly related to the function of the lungs [7–9,12,14]. Other studies, directed towards understanding the mechanisms behind VOC synthesis, sample the headspace of LC cell lines such as A549, H1299, H1975, HCC827, H226, H520, H460, and H526 [8,34,37,72,73]. Furthermore, VOCs have been sampled from tumor tissue samples as well as from the urine of mice with experimental tumors [8,9,28].

Table 1 presents VOCs found by a minimum of 2 different research groups using 2 or more different systems of study (i.e., cell- or tumor-based, animal-based, or human-based systems). Out of the 116 VOCs reported to show significant difference (*P* < 0.05) between LC patients and healthy controls in 11 different studies, only 19 VOCs met the criteria.

**Table 1.** VOCs found to show significant difference between lung cancer and healthy control in 2 or more studies using 2 or more study systems

Chemical group	Compound	CAS no.	Papers found in
Alcohol	Ethanol	64-17-5	[8,12]
Aldehyde	Propanal	123-38-6	[14,62]
	Butanal	123-72-8	[9,14]
	Pentanal	110-62-3	[9,14]
	Hexanal	66-25-1	[9,14,62]
	Nonanal	124-19-6	[12,14,34,61,62]
Aromatic aldehyde	Benzaldehyde	100-52-7	[12,14,28,37,62]
Alkane	Acetone	67-64-1	[9,12,62]
Hydrocarbon	Octane	111-65-9	[8,12]
	Dodecane	112-40-3	[7,12,34]
	Tridecane	629-50-5	[12,61]
Aromatic hydrocarbons	Dimethylbenzene isomers (*m*-xylene, *o*-xylene, *p*-xylene)	108-38-3, 95-47-6, 106-42-3	[9,62]
	Toluene	108-88-3	[7,9,18,37]
	Styrene	100-42-5	[12,37,62]
	1,2,4-Trimethylbenzene	95-63-6	[12,62]
	Ethylbenzene	100-41-4	[9,12,62]
Ketone	Acetophenone	98-86-2	[12,34]

Tsou et al. and Filipiak et al. observed higher levels of ethanol in LC patients [12], tumor tissues, and A549 cancer cell lines [8] when compared to healthy patients, healthy lung tissue, and control medium. Furuhashi et al. [72] suggest that alcohols may arise in mammalian cells through the reduction of aldehydes. The accumulating mutations inside cancer cells lead to excess reactive oxidative species (ROS), which could lead to membrane degradation, which causes lipid peroxidation [12]. Aldehydes, among other toxic compounds, are produced by lipid peroxidation. The enzyme ADH (alcohol dehydrogenase) facilitates a reversible chemical reaction to convert an aldehyde into an alcohol [12]. However, ADH has been shown to have low activity in LC tissues taken from patients [74]. This discrepancy highlights the current knowledge gap regarding the biochemical pathways involved in VOC production. While groups hypothesize that an upregulation of ADH causes an upregulation in emitted alcohol VOCs, this hypothesis is not supported by in vitro studies [74].

Li et al. [14] found increased levels of propanal, butanal, pentanal, hexanal, and nonanal in the exhaled breath of LC patients compared to healthy controls by targeting the capture of aldehydes on a paper substrate anchored with a probe molecule. Jia et al. [73] also saw increased levels of propanal in adenocarcinoma cell lines A549 and HCC827, the squamous carcinoma cell line H520, the large cell carcinoma cell line H460, and the normal epithelial cell line small airway epithelial cells (SAEC) each compared to their respective control medium. Gashimova et al. [9] saw an increase in the level of butanal in exhaled breath and tumor tissue samples from LC patients but saw a decrease in pentanal, which opposes the finding of Li et al. [14]. Jia et al. also observed a decrease in nonanal in H526 small cell carcinoma cells compared to control media, which again contradicts the findings by Li et al. However, Furuhashi et al. [72] observed an increase in nonanal in A549 two-dimensional and three-dimensional cell cultures, and Serasanambati et al. [34] observed an increase in nonanal in H1299 cells, corresponding to the findings of Li et al. Serasanambati et al. highlight that H1299 is a p53 null-type cell line and hypothesize that the absence of gene hampering metabolic regulation in the cell may cause the increase in nonanal for this cell type. Interestingly, Serasanambati et al. also investigated the change in concentration of VOCs released by bulk cells in a time-dependent manner and found that nonanal levels increase at 24 h but decrease after 48 h. This observed change in concentration demonstrates the influence of growth progression on the biosynthesis of certain compounds and emphasizes a point that should be addressed in a future study. Benzaldehyde was reported to decrease in A549 cells, H1299 cells, H1975 cells [34], H520 cells, and SAEC cells [73], and increase in A549 cells, H460 cells [73], and the breath of LC patients. The fact that Serasanambati et al. saw a decrease in the same VOC in the same cell line where Jia et al. saw an increase emphasizes the need to evaluate VOC trends at all stages, from the single cell to the bulk cell, to animal studies, and finally breath studies. In vitro experiments must be conducted with a focus on biochemical pathways in order to understand and validate the VOCs being reported as potential biomarkers of LC from in vivo studies. Without understanding the origins of the VOC biomarkers reported, they will continue to be of limited use in a research setting because they are not reliable testing strategies. Concerning the overall trends of the aldehydes included in Table 1, a majority of studies reported increased levels. This observed increase in aldehyde levels is in direct contrast with the reported upregulation of aldehyde dehydrogenase (ALDH) in LC patients and cells [74–78].

The alkane acetone was found to be increased in H460 and SAEC cells compared to their respective control media [73], tumor tissue samples [9], and the exhaled breath of LC patients [12]. Oxidative stress affects the release of alkanes since their production via lipid peroxidation relies on the peroxidation of polyunsaturated fatty acids by ROSs; therefore, increases in oxidative stress causes increases in ROSs and ultimately increased levels of alkanes [73]. It is also possible that these alkanes will be further oxidized by ADH or ALDH into aldehydes or carboxylic acids, respectively, possibly explaining the increased levels of aldehydes noted earlier. Gashimova et al. also point out that the levels of acetone in the exhaled breath can be influenced by breath holding, and closely monitored their sampling procedure to prevent this influence; however, they conclude that acetone concentrations reported in some cases may be ambiguous due to the influence of breath sampling procedures [9]. Without a standard protocol for the collection and analysis of samples from a variety of biofluids, the impact of confounding factors will continue to cause significant obstacles in understanding the origins of emitted VOCs. If potential VOC biomarkers cannot be attributed to endogenous reactions, their use in the VOC signature of a disease can cause misdiagnosis, the exact problem VOC biomarkers are attempting to solve.

The generation of saturated hydrocarbons can be attributed to oxidative stress in the same way as alkanes. They are the result of the peroxidation of polyunsaturated fatty acids by ROSs [9,73].

Levels of styrene have been observed at both increased [37] and decreased [73] levels in various cell lines. Toluene overall had a trend of increased levels in A549 cell culture [72], EML4-ALK cell culture [37], lung tumor tissue samples, and the breath of LC patients [9]. The consistency of the trends of toluene seems promising, but, as Peled et al. point out, aromatic compounds, specifically benzene derivatives, are typically considered to be of exogenous origin. All of the aromatic hydrocarbons reported in Table 1 are benzene derivatives and may reflect VOCs that had been absorbed into the lung tissues of the donors from which the cell lines were established. Particularly for LC cell lines, the smoking habits of the donor can affect the VOCs absorbed into the lung tissue before immortalization [73]. As mentioned above, VOC biomarkers must be connected to endogenous origins in order to be clinically relevant. If an exogenous VOC is reported as a biomarker of disease, using this VOC signature may result in misdiagnosis based on confounding factors, leading to unnecessary expenses to confirm a diagnosis with another method.

#### Breast cancer

BC can be categorized into 5 molecular subgroups (Luminal A, Luminal B, Triple-negative, human epidermal growth factor receptor 2 (HER2)-enriched, and Normal-like) through the use of immunohistochemistry (IHC), gene mutation analysis by DNA sequencing, and chromosomal alterations by fluorescence in situ hybridization to evaluate estrogen receptor (ER), progesterone receptor (PgR), and HER2 protein expression [44,79]. While the use of gene expression profiling is widespread, it remains limited due to high costs and technical difficulties [80]. Screening techniques for BC currently include self-examination, mammography, ultrasound, MRI, and digital breast tomosynthesis (DBT); however, these techniques have relatively low sensitivity and specificity as the accuracy of these methods relies largely on the experience of the physician and the tumor’s histopathologic features [18,36,44]. Ductal carcinoma in situ (DCIS) is the preinvasive stage of BC, and there are currently no established screening methods for DCIS of the breast [18]. Many VOC BC studies focus on discriminating between the molecular subtypes of BC, using cell lines with different BC classifications in order to find biomarkers suitable for patient risk assessment [80]. All BC cell lines used in studies included in this review and their classification are shown in Table 2. Woollam et al. [27] used female BALB/c mice injected with 4T1.2 mammary tumor cells either in the mammary pad, to model localized cancer, or into the iliac artery, to model metastasized BC. Urine from the mice was collected 18 days after injection for VOC analysis. While all BC VOC studies utilizing human breath [7,16,18,80] aimed to discriminate between patients with BC and healthy controls, Barash et al. went the further step to discriminate between patients with DCIS, benign, and malignant lesions and Zhang et al. worked to discriminate not only between patients with DCIS or lymph node metastasis (both positive and negative status) but also from those with gastric cancer, as HER2 is also present in this disease [18,80].

**Table 2.** Molecular subtypes of BC cell lines

Classification	Cell line	Number	ER	PgR	HER2	Paper
Triple negative	MDA-MB-231	1	−	−	−	[44,36,68]
Luminal A	MCF-7	2	+	+	−	[44,36,68]
	T-47D	3	+	+	−	[36]
Luminal B	BT-474	4	−	+	+	[44]
	ZR-75-1	5	+	+	+	[44]
HER2-enriched	SKBR-3	6	−	−	+	[44]

Table 3 presents VOCs found by a minimum of 2 different research groups using 2 or more different systems of study (i.e., cell- or tumor-based, animal-based, or human-based systems). Out of the 130 VOCs reported to show significant difference (*P* < 0.05) between BC and control in nine different studies, only 11 VOCs met the criteria.

**Table 3.** VOCs found to show significant difference between breast cancer and healthy control in 2 or more studies using 2 or more study systems

Chemical group	Compound	CAS no.	Papers found in
Alcohol	2-Ethyl-1-hexanol	104-76-7	[18,36,44,68,69]
Ester	Ethyl acetate	141-78-6	[36,69]
Ketone	2-Pentanone	107-87-9	[27,36]
	Acetone	67-64-1	[36,69]
Cyclic ketone	Cyclohexanone	108-94-1	[18,36]
Aromatic hydrocarbon	Styrene	100-42-5	[36,69]
	1,3-Dimethylbenzene (*m*-xylene)	108-38-3	[36,69]
	Ethylbenzene	100-41-4	[36,69]
Aromatic aldehyde	Benzaldehyde	100-52-7	[27,36]
Phenol	Phenol	108-95-2	[18,36]
	2,4-Di-*tert*-butylphenol	96-76-4	[27,36]

The alcohol 2-ethyl-1-hexanol (also referred to as 2-ethylhexanol) was the most commonly reported VOC for BC. It was found to be upregulated in BC cell lines 1, 2,4, 5, and 6 (cell lines corresponding to number can be found in Table 3) compared to the respective control medium by Lavra et al.; upregulated in BC cell lines 1, 2, and 3 compared to normal epithelial cell line HMEC by Silva et al.; upregulated in BC cell lines 1 and 2 compared to normal cell line fibroblast CCD-1095Sk by Huang et al.; and upregulated in the breath of cancer patients compared to healthy controls by Barash et al. Zhang et al. also reported that 2-ethyl-1-hexanol was able to discriminate between the breath of patients with DCIS and healthy controls, patients with lymph node metastasis-negative BC and healthy controls, and patients with lymph node metastasis-positive BC and healthy controls but did not report if 2-ethyl-1-hexanol was up- or downregulated. Without reporting the trends (i.e., up or downregulated) of reported VOCs, no hypothesis can be made about their origins, which undermines the effort to transition VOC biomarkers to clinical use. Silva et al. hypothesize that higher alcohols (i.e., alcohols containing more than 2 carbons) are endogenously generated as hydrocarbon metabolism by-products. ADH (discussed in the “Lung cancer” section) and cytochrome P450 activities are known to metabolize hydrocarbons into aldehydes or ketones with alcohol by-products [36,80]. Cytochrome P450 is known to be overexpressed in BC cells and may enhance the activity of ROS molecules, which further enhance the activity of a varied group of oxidase enzymes [36,80]. Interestingly, Larva et al. noted that 2-ethyl-1-hexanol is 1 of the 3 VOCs they observed that had the same behavior in the headspaces of both ER- and PgR-positive cell lines. They hypothesized that these VOCs could be associated with specific receptor-related metabolic pathways in cells with positive expression of these IHC markers [44].

Silva et al. [36] found ethyl acetate in significantly increased quantities in the headspace of the T-47D cell line. This finding is noteworthy since ethyl acetate was not found in significant quantities in the headspace of the MCF-7 cell line, which is also classified as Luminal A with the same IHC markers. Barash et al. [80] also reported an increased level of ethyl acetate in the breath of BC patients compared to healthy controls.

The reporting of benzaldehyde as a VOC biomarker of BC has been questioned since it has been commonly found to be solely present in controls [36,44]. While Silva et al. still reported benzaldehyde as a VOC biomarker, Larva et al. excluded the compound in their analyses based on this finding. Woollam et al. reported that benzaldehyde was upregulated in BC when compared to controls and downregulated in metastatic BC when compared to localized BC in their mouse model. This discordance emphasizes the need to standardize both the collection and analysis of VOC samples. When determining which VOCs may be potential biomarkers for disease, there should be a standard for how those VOCs correlate between healthy and diseased individuals. If the VOC should be present in both healthy and diseased individuals, an approximate concentration change should be determined and validated with in vivo experiments. Pathways that could contribute to this concentration change can be identified and studied individually. If the VOC is thought to be a product of disease-specific interactions, or the degradation of the VOC is a result of the disease, the up- or downregulation of the VOC should be determined in order to characterize the biochemical pathways involved in the diseased model.

#### Colorectal cancer

The early stages of CRC often present only mild symptoms or are asymptomatic, making early diagnosis difficult as symptoms are easily ignored [31]. The most accurate and reliable method for CRC diagnosis is a colonoscopy and biopsy, but the high cost and invasiveness of the procedure, along with the risk of iatrogenic colonic perforation, prevent its use as a large-scale CRC screening tool [30,31,81]. The fecal immunochemical test and the fecal occult blood test are the most widely used noninvasive screening methods for CRC. While the fecal immunochemical test exhibits good specificity for CRC, its sensitivity is low and varies from patient to patient [31]. The fecal occult blood test exhibits both low sensitivity and specificity for CRC, with only a 10% positive predictive value [30,81]. Specific serum tumor biomarkers, such as carcinoembryonic antigen and carbohydrate antigen 19-9, have been used in clinical diagnosis of CRC, but both antigens are also biomarkers for other types of nongastrointestinal tumors, making them less effective in screening for CRC [30,31,81]. As a result of the poor diagnostic methods for CRC, VOC studies have focused on identifying promising biomarkers of CRC by comparing emitted VOCs from the breath [7,19,81], blood [30], urine [24], and feces [39] of CRC patients compared to healthy controls. The VOCs emitted by the LS174T human colon cancer cell line compared to those emitted by the HCoEpiC human normal intestinal epithelial cell line were also studied by Liu et al. [31] in order to identify CRC-specific VOCs without the confounding factors present in exhaled breath. To this extent, Liu et al. [31] also cultured tumors in female BALB/C nude mice, which were then excised and transplanted into male Sprague Dawley rats whose blood was sampled at various time points to model colon cancer.

Table 4 presents VOCs found by a minimum of 2 different research groups using 2 or more different systems of study (i.e., cell- or tumor-based, animal-based, or human-based systems). Out of the 53 VOCs reported to show a significant difference (*P* < 0.05) between CRC and control in 6 different studies, only 3 VOCs met the criteria.

**Table 4.** VOCs found to show significant difference between colorectal cancer and healthy control in 2 or more studies using 2 or more study systems

Chemical group	Compound	CAS no.	Papers found in
Alcohol	2-Ethyl-1-hexanol	104-76-7	[19,30,31]
Aldehyde	Decanal	112-31-2	[19,31]
Phenol	Phenol	108-95-2	[31,70]

Liu et al. [31] reported lower levels of 2-ethyl-1-hexanol in (a) the CRC cell line LS174T compared to the normal intestinal epithelial cell line HCoEpiC, (b) the LS174T cell line compared to control medium, and (c) the LS174T cell line compared to the same cell line treated with the chemotherapeutic arsenic trioxide (ATO). Wang et al. [30] reported similar trends of lower levels of 2-ethyl-1-hexanol in the blood of CRC patients compared to healthy controls. Altomare et al. [19], however, reported higher levels of 2-ethyl-1-hexanol in the breath of CRC patients compared to healthy controls. As the accumulation of ROS, a common trait of many cancers, can lead to the production of hydrocarbons, whose oxidation is associated with the production of alcohols, increased levels of alcohols are normally associated with VOC cancer experiments. As 2-ethyl-1-hexanol was identified in both CRC and control groups by Liu et al., they hypothesize that the decomposition of the exogenous plasticizer di(2-ethylhexyl)phthalate to 2-ethyl-1-hexanol could be responsible for the presence of this alcohol in their in vitro experiments. Wang et al. hypothesize that the lower levels of 2-ethyl-1-hexanol in the blood of CRC patients could be due to the consumption that occurs during tumor cell proliferation.

Liu et al. reported lower levels of decanal in the LS174T cell line compared to control medium and the LS174T cell line compared to the same cell line treated with the chemotherapeutic ATO. Again, Altomare et al. reported an opposing trend, finding higher concentrations of decanal in the breath of CRC patients compared to healthy controls. While neither research group hypothesizes the mechanisms underlying these trends, the levels of aldehydes are normally related to the levels of ROS in the microenvironment and their effects on lipid peroxidation. If a tumor of a cancerous cell line has lower activity in terms of proliferation and metabolism, there will be less oxidative stress and lipid peroxidation, leading to lower levels of emitted aldehydes.

Liu et al. reported lower levels of phenol in the LS174T cell line compared to control medium and the LS174T cell line compared to the same cell line treated with the chemotherapeutic ATO. De Vietro et al. [81], on the other hand, reported higher levels of phenol in the breath of CRC patients compared to healthy controls. De Vietro et al. state that phenols are not produced by human enzymes, but instead are unique colonic bacterial metabolites generated during the bacterial degradation of tryptophan. Phenol and other colonic bacterial metabolites are of special interest in CRC VOC studies since the role of the gut microbiota has recently been believed to be particularly relevant in the carcinogenesis of CRC [81]. This finding highlights an important aspect of in vitro VOC studies that has not been fully explored: how cell–cell and cell–matrix interactions affect VOC production and collection. The limitation of traditional cell cultures to model these interactions can be overcome through the use of microfluidic cell culture models to reflect the organ of study [82–84]. Furthermore, VOCs preliminarily identified in in vitro studies should be validated using both animal and human models.

#### Prostate cancer

PC is usually detected through a combination of digital rectal examination and serum prostate-specific antigen (PSA) measurements, followed by a transrectal ultrasound-guided prostate biopsy [23,25,35]. While elevated levels of PSA (i.e., >4.0 ng/ml) are associated with PC, they are also associated with benign conditions such as prostatitis and benign prostatic hyperplasia (BPH), which are common in the elderly [23,35]. Consequently, PSA screening for PC has low sensitivity and can lead to unnecessary prostate biopsies, which is an invasive and expensive procedure.

Table 5 presents the single VOC out of the 46 reported by 4 different research groups to show significant difference (*P* < 0.05) between PC and controls using 2 or more different systems of study (i.e., cell- or tumor-based, animal-based, or human-based systems). It should be noted that this VOC was reported by the same research group in 2 distinct studies using 2 distinct experimental models at different time points. No VOCs reported by different research groups were found to be the same.

**Table 5.** VOCs found to show significant difference between prostate cancer and healthy controls using 2 or more different systems of study

Chemical group	Compound	CAS no.	Papers found in
Cyclic ketone	Cyclohexanone	108-94-1	[25,35]

Interestingly, Lima et al. reported decreased levels of cyclohexanone in the medium of PC cell lines 22RV1, PC3, DU145, and LNCaP compared to the normal prostate epithelial cell line PNT2 [35] but reported increased levels of cyclohexanone in the urine of PC patients compared to healthy controls [25]. It should be noted that cyclohexane was only reported at significantly altered levels when VOC extraction was performed at pH 2 rather than at pH 7, the natural pH of prostate cell culture medium. Lima et al. chose to perform VOC extraction at an acidic pH value as well as the natural pH of the medium since it is known that the tumor microenvironment of PC is acidic in order to promote tumor progression and metastasis [35]. This decision is particularly thought-provoking as the discriminant VOCs reported for each pH were not the same, highlighting the importance of conducting PC VOC studies at different pH values to obtain a comprehensive picture of the VOCs emitted by PC cells in different microenvironments. As with the CRC studies, understanding and mimicking the microenvironment of the organ of study is critical to obtaining physiologically relevant VOC profiles of disease. Without these cell–cell and cell–matrix interactions, in vitro VOC models are limited in their ability to provide relevant VOCs to investigate.

During their VOC study using urine as the sample biofluid, Lima et al. also computed Spearman’s correlation indexes using all VOCs found to be significantly different between PC patients and healthy controls to identify VOCs either in the same metabolic pathway or under a common regulatory mechanism. They reported strong positive correlations for both hexadecane and 3-phenulpropionaldehyde with cyclohexanone [25]. Lima et al. hypothesize that increased levels of ketones, such as cyclohexanone, can be explained by increased fatty acid β-oxidation and protein metabolism, which leads to overproduction of ketones.

### Neurodegenerative diseases

AD and PD are some of the most prevalent neurodegenerative disorders. AD is a progressive neurodegenerative disease of the central nervous system where patients suffer from a decline in memory, language, and other cognitive abilities due to damaged neurons in the parts of the brain involved in memory, such as the hippocampus, and higher-level processes, such as the cerebral cortex. Late-stage AD is characterized by amyloid beta plaques and neurofibrillary tangles [10,22,61], and measurements of amyloid beta, tau protein, and phosphorylated tau protein in cerebrospinal fluid (CSF) have become biomarkers for pathologic confirmation [20,61]. In contrast, PD is associated with progressive death of dopaminergic neurons in the substantia nigra pars compacta and widespread dopamine depletion resulting in resting tremors, bradykinesia, rigidity, and postural instability [10,32]. PD is mainly diagnosed on clinical signs [61]; however, by this time, more than 50% of dopaminergic neurons and 75% to 80% of striatal dopamine are already lost [32]. While CSF seems the optimal biofluid in searching for AD and PD biomarkers due to its proximity to the pathology, CSF testing is invasive, costly, and risky to the patient. Finding appropriate, noninvasive biomarkers of AD and PD could enable early identification of characteristic pathologic alteration in genetically at-risk groups and thus allow treatment before neuronal damage develops.

This section summarizes recently published data regarding AD and PD VOC studies, reports biomarkers found, and explores possible underlying biochemical pathways.

#### Alzheimer’s disease

AD VOC studies have focused on the testing of exhaled breath in patients with AD [6,10,20,61,85] as well as the exhaled breath from human apolipoprotein E4 (APOE4) knock-in rats on high-fat/high-sucrose (HFHS) diets [22]. APOE has been recognized as a major risk factor contributing to the dementia that defines AD due to its role in delivering cholesterol and other complex lipids to neurons for membrane maintenance, neurogeneration, and repair [22]. The APOE4 isoform specifically interacts with the mitochondria to reduce respiratory capacity and function, which ultimately compromises energy use [22]. Individuals carrying one allele of the APOE4 isoform are twice as likely as noncarriers to develop AD, while individuals carrying 2 alleles are 7 times more likely than noncarriers to develop AD [22]. As the progression of AD can be categorized into 3 phases—preclinical, mild cognitive impairment, and clinical—studies have also focused on distinguishing among these phases in order to circumvent the heterogeneity of AD patients that often prevents early diagnosis [6].

Table 6 presents VOCs found by more than one research group. Three of these VOCs [2,3-dimethylheptane, 2,2-dimethylpropanoic acid, and butylated hydroxytoluene (BHT)] were reported in different systems of study, while one VOC (1-butanol) was only reported for human breath.

**Table 6.** VOCs found to show significant difference between AD and healthy controls

Chemical Group	Compound	CAS No.	Papers found in
Alcohol	1-Butanol	71-36-3	[6,58]
Hydrocarbon	2,3-Dimethylheptane	3074-71-3	[10,22]
Short-chain fatty acid	2,2-Dimethylpropanoic acid	75-98-9	[10,22]
Synthetic phenolic compound	Butylated hydroxytoluene	128-37-0	[10,22]

The exact mechanisms underlying AD pathogenesis have not been completely revealed, but there is evidence that ROS plays a role in the pathophysiological cascade that leads to AD [6,20]. While resting levels of ROS reactivity contributes to cellular and tissue damage, once ROS generation exceeds the endogenous ability to destroy them, oxidative stress develops and causes damage to the cells, proteins, nucleic acids, sugars, and even lipids [85]. ROS-induced lipid peroxidation is thought to be a contributing factor in the progression of AD as it causes neuronal damage and cell death [22]. There is some evidence suggesting that dysfunction in mitochondrial metabolism causes this increased production of ROS [6].

The 3 compounds found by Tisch et al. and Emam et al. [22] are not known to be directly linked to the dyslipidemia and lipid peroxidation associated with AD. 2,3-Dimethylheptane is known to be one of many VOCs generated by household fungus. 2,2-Dimethylpropanoic acid is used in a clinical setting to increase the bioavailability of antibiotics [22]. While BHT occurs naturally in bacteria and some plants, it is more commonly used as an antioxidant additive to foods and cosmetics [22]. Furthermore, BHT can be taken as a nutritional supplement to potentially prevent dementia [22]. While these uses of BHT may lead to the assumption that its generation is dependent on exogenous origins, BHT was also detected in the breath of APOE4 rats on an HFHS diet. This raises the following question: Can these compounds be generated by an HFHS diet alone, or do they require the accompanying APOE4 genotype? Emam et al. give 3 possible explanations for BHT generation in APOE4 rats on HFHS diets: (a) BHT production in APOE4 genotype rats on HFHS diets relies on an unknown metabolic pathway, (b) the BHT sensor detects a similar organic phenolic hydrocarbon, or (c) BHT is not a biomarker of AD. They conclude that the third possibility is the most probable. Ultimately, this line of questioning is important for identifying and understanding the confounding factors present in VOC studies as they can alter the results and their physiological relevancy. If diet is found to significantly affect the VOCs produced by animal models, the effect of diet on VOCs derived from human-based studies should be more thoroughly investigated before potential VOC biomarkers are reported.

Another VOC possibly influenced by the diet of AD patients is acetone. Tiele et al. note that increased levels of acetone in the exhaled breath of AD patients could be associated with region-specific declines in brain glucose metabolism. Ketone bodies, such as acetone, acetoacetate, and beta-hydroxybutyrate, are produced from fat stores when glucose is unavailable. This deficit of glucose occurs during periods of fasting or keto diet, and nearly half of mild-AD subjects suffer a loss of appetite, which could lead to increased levels of acetone in the exhaled breath [6].

#### Parkinson’s disease

PD VOC studies have tested human breath samples [10,61], human sebum samples [41,42], rat breath samples [86], rat blood samples, and rat striatum tissue samples [32]. Interestingly, the decision to collect sebum samples was guided by Joy Milne, an individual with an extremely sensitive sense of smell that has allowed her to identify a distinct “PD odor” in her husband who was diagnosed with PD in 1986 [41]. Sebum is a waxy, lipid-rich biofluid that is excreted by the sebaceous glands in the skin [41]. Preliminary tests indicated that this PD odor was most prominent in areas of high sebum production, particularly the upper back and forehead rather than the armpits. Overproduction of sebum is known as seborrhea and is a known nonmotor symptom of PD [41]. The animal model for PD used by Khatib et al. and Tisch et al. consists of rats treated with 250 μg of the neurotoxin 6-hydroxydopamine, which results in partial (55% ± 5%) lesion of dopaminergic neurons in the nigrostriatal pathway [32,86]. This degree of dopaminergic lesion represents the degree at which the first motor symptoms associated with PD are detected in both rats and humans [32,86]. As rats and humans have similar responses to graded striatal dopaminergic depletion, the rat model allows for precise determination of the degree of dopaminergic cell loss and provides a physiological system devoid of complications caused by drug treatment and variable dietary conditions [86]. However, since PD develops over decades in a human rather than the 5 weeks that the rats are injected with 6-hydroxydopamine, the rat model is limited in its representation of the PD pathology.

The possible influence of medication on the chemical composition of VOCs produced is an important consideration. Tisch et al. address this issue by ensuring that the medication taken by the study group was sufficiently diverse in order to reduce the risk of false-positive distinction due to specific medications. The PD rat model allows a system of study independent of administered medication [86]. Trivedi et al. showed that, in sebum samples, there were no observable significant differences between the VOC profiles of PD participants on medication and those that were drug naïve. This insignificant change in the sebum of PD patients due to changes in medication highlights the potential of this biofluid for biomarker research.

Table 7 presents 2 VOCs reported in 2 separate studies, conducted by the same group, in different systems. Tisch et al. first reported the breath print of presymptomatic PD in the rat model [86] and a year later expanded the study to 57 human volunteers [10].

**Table 7.** VOCs found to show significant difference between PD and healthy controls

Chemical group	Compound	CAS no.	Papers found in
Aromatic hydrocarbon	Styrene	100-42-5	[10,72]
Alkane	5-Ethyl-2-methyl-octane	62016-18-6	[10,72]

Styrene has been linked to DNA damage and is a known environmental toxin [10]. While styrene is typically attributed to exogenous origins, it was also observed in increased amounts in the breath of rats with an asymptomatic nigrostriatal dopaminergic lesion compared to control animals kept in the same environment and receiving the same food [86]. This observation could indicate that styrene is indeed endogenously produced; however, in vitro studies free from dietary confounding factors should be conducted to investigate its origins.

The methylated alkane, 5-ethyl-2-methyl-octane, could be a by-product of the phospholipids associated with the breakdown of the cell membrane [86]. However, as these phospholipids are not unique to the brain, 5-ethyl-2-methyl-octane could result from a general increase in cell death, and not specifically dopaminergic neuron death [86].

### Other noninfectious diseases

#### Epilepsy

Epilepsy is a chronic disorder of the brain, characterized by a predisposition to generate seizures, which are a transient occurrence of abnormal synchronizations of neuronal activity in the brain [21,29]. There are many forms of epilepsy and initial management starts with understanding the seizure type [21,29]. Diagnosis of epilepsy following a first seizure relies on a combination of clinical information and diagnostic tests, such as electroencephalograms and MRIs; despite diagnostic guidelines being in place, diagnosing epilepsy is complicated and time-consuming [21]. Uncontrolled epilepsy is a burden on the patients and caregivers as it worsens quality of life and increases the chance of both physical and psychiatric comorbidities [29]. VOCs represent a potential, new diagnostic tool for faster, easier, and more sensitive diagnosis of epilepsy. Of the 2 epilepsy VOC studies included in this review, Dartel et al. focused on using an eNose for the discrimination of breath profiles of epilepsy patients and epilepsy-free controls while Fujita et al. [29] focused on identifying VOC biomarkers associated with epilepsy by sampling the urinary volatilome of an amygdala-kindled mouse model. The mouse model consists of conscious, unrestrained mice that receive a biphasic square wave pulse into the basolateral amygdala one a day for approximately 3 weeks in order to model human mesial temporal lobe epilepsy, a form of epilepsy exhibited by half of all adult epilepsy patients [29].

Table 8 presents the 13 VOCs (AUC > 0.8) reported as potential epileptic biomarkers in mice by Fujita et al., excluding the 2 unknown VOCs reported that are not found in humans.

**Table 8.** VOCs found to show significant difference between epileptic individuals and healthy controls

Chemical group	Compound	CAS no.
Amine	Trimethylamine	75-50-3
Aromatic compound	2-Acetyl-1-pyrroline	85213-22-5
Ketone	2-Butanone	78-93-3
	2-Pentanone	107-87-9
	2-Heptanone	110-43-0
	Acetophenone	98-86-2
Nitroalkane	Nitromethane	75-52-5
Pheramore	3,4-Dehydro-*exo*-brevicomin	62255-25-8
Pyrrole	2-Acetylpyrrole	1072-83-9
Sulfide	Dimethyl disulfide	624-92-0
	Dimethyl trisulfide	3658-80-8
	2,3,5-Trithiahexane	42474-44-2
Thiol	Methanethiol	74-93-1

Methanethiol, disulfide, dimethyl, dimethyl trisulfide, 2,3,5-trihiahexane, 2-acetyl-1-pyrroline, 2-acetylpyrrole, trimethylamine, methane, nitromethane, and 2-heptanone decreased in the urine of mice following kindled seizures. 2-Butanone and 2-pentanone increased in the urine of mice following kindled seizures. Methanethiol is naturally formed in mammalian tissues and by the microbiota in the intestinal tract [44]. In the gut flora, methanethiol is normally derived from methionine and oxidizes to dimethyl disulfide and dimethyl trisulfide in the presence of pyridoxal phosphate. 2,3,5-Tritiahexane is a pheromone released by male mice. The thermal degradation products of methionine and photolysis of dimethyl trisulfide lead to the exogenous production of this pheromone, and as epileptic seizures are associated with decreased polysulfuration, they lead to decreased levels of 2,3,5-tritiahexane in the urine of mice following seizures. The biosynthesis of 2-acetyl-1-pyrroline is inhibited by the enzyme betaine aldehyde dehydrogenase (Badh2), and mutations in the human gene ALDH7A1, the homolog of Badh2, cause pyridoxine-dependent epilepsy. Activation of ALDH7A1 in epileptic mice may lower the stability of 2-acetyl-1-pyrroline and 2-acetylpyrrole, which would result in the decreased levels observed following a seizure. Trimethylamine functions as a pheromone for both male and female mice and the microbiota metabolizes both phospholipid PC and choline to form trimethylamine. 2-Butanone, 2-pentanone, and 2-heptanone are methyl ketones, which are mainly derived by the fatty acid beta-oxidation network and glycolysis reactions in bacteria. 2-Butanone and 2-pentanone, specifically, have been shown to be produced from glucose by *Klebsiella pneumoniae* in the gut flora. It is of note that trimethylamine, methanethiol, 2-butanone, disulfide, dimethyl, 2-heptanone, dimethyltrisulfide, and 2-aceptylpurrole were found in significant quantities in the diet of the mice, indicating that these 8 VOCs may be absorbed from the diet.

While Dartel et al. did not report specific VOCs, they tested for the influence of antiepileptic drugs (AEDs) on the epilepsy breath profile and found that medication usage had a large impact on the number of false positives and false negatives obtained by their model. This suggests that patient breath profiles may be mainly based on the usage of various AEDs and future studies will need to be conducted with patients who do not yet take AEDs in order to avoid this influence and increase the diagnostic capabilities of their epilepsy VOC model.

#### Autism

Autism spectrum disorders (ASDs) are a group of neurodevelopmental dysfunctions, resulting in an impaired ability to communicate and interact [26]. The pathogenesis of ASDs is complex and can be associated with genetic disorders and/or inborn errors of metabolism, pre- and/or postnatal exposure to chemicals or viruses, dysfunctional immune systems, and more [26]. As recent studies have found alterations in host metabolism and gut microbiota in ASD individuals, which would result in altered metabolite profiles, it is natural that the search for biomarkers of ASDs would transition to the study of VOCs. Cozzoline et al. aimed to identify putative VOC biomarkers that could discriminate between ASD children and healthy controls by using urine as the sample biofluid. To take into account the structural diversity of VOCs present in urine, they studied samples of urine under both acidic pH (pH 1 to 2) conditions, to improve extraction of acids and sulfur compounds, and alkaline pH (pH 12 to 14) conditions, to improve extraction of alcohols, amines, ketones, and heterocyclic compounds. The 8 VOCs found under acidic conditions and the 7 VOCs found under alkaline conditions are listed in Table 9.

**Table 9.** VOCs found to show significant difference between ASD individuals and healthy controls

Chemical group	Compound	CAS no.	Acidic or alkaline conditions
Aldehyde	3-Methylbutanal	590-86-3	Acidic
	2-Methylbutanal	96-17-3	Acidic
Alcohol	Ethanol	64-17-5	Acidic
Alkane	Hexane	110-54-3	Acidic
Furan	2-Methylmercaptofuran		Acidic
	2-Pentylfuran	3777-69-3	Acidic
Isoxazole	Isoxazole	288-14-2	Alkaline
Ketone	3-Methylcyclopentanone	1757-42-2	Acidic
	Acetophenone	98-86-2	Alkaline
	2-Heptanone	110-43-0	Alkaline
Pyrazine	2-Methylpyrazine	109-08-0	Alkaline
	2,3-Dimethylpyrazine	5910-89-4	Alkaline
Pyridine	3-Ethylpyridine	536-78-7	Alkaline
Sulfide	Dimethyl trisulfide	3658-80-8	Acidic
	Methoxyphenyl oxide		Alkaline

3-Methylcyclopentanone, 2-methylbutanal, 3-methylbutanal, hexane, 2-methylpyrazine, 2,3-dimethylpyrazine, and isoxazole were found to have higher levels in the urine of autistic children than controls. Ethanol, 2-methylmercaptofuran, 2-pentylfuran, dimethyl trisulfide, methoxyphenyl oxide, 3-ethylpyridine, acetophenone, and 2-heptanone were found to have a lower concentration in the urine of autistic children than controls. They hypothesize that since 3-methylbutanal is involved in the catabolism of leucine, an upregulation of the enzymes involved in the metabolic pathways associated with the catabolism of leucine in autistic children could result in the elevated levels observed. Furthermore, as discussed in the “Lung cancer” section, aldehydes are produced by lipid peroxidation, and as lipid peroxidation is elevated in autism, the higher concentrations of 2-methylbutanal and 3-methylbutanal could be related to lipid peroxidation and oxidative stress in ASDs. Low intestinal carbohydrate-digestive enzyme activity is a commonly observed abnormality in autistic children. As furan derivatives can be generated by the enzymatic degradation of dietary carbohydrates, the lower levels of 2-methylmercaptofuran and 2-pentylfuran observed in autistic children compared to controls could be attributed to this enzymatic degradation downregulation.

#### Arthritis

There are 9 types of arthritis, according to the Mayo Clinic, with osteoarthritis and rheumatoid arthritis (RA) being the most common [59]. While osteoarthritis is associated with the wearing away of the cartilage that coats the joint bones, RA is a systemic immune-mediated inflammatory disease associated with the immune system attacking the lining of the joints [59]. Early diagnosis is key in arthritis as delaying therapeutic intervention can lead to irreversible damage and the destruction of cartilage and bone within the joints. Brekelmans et al. [15] conducted a case–control study to investigate whether an eNose could discriminate between the exhaled breath from RA patients, psoriatic arthritis (PsA) patients, and healthy volunteers. PsA is a form of arthritis that can affect those with psoriasis. They also performed GC-MS analysis of exhaled breath in order to identify any disease-specific VOCs. A total of 7 VOCs were identified by GC-MS, with 3 being unknown compounds. The 4 known compounds reported are listed in Table 10.

**Table 10.** VOCs found to show significant difference between individuals with arthritis and healthy controls

Chemical group	Compound	CAS no.
Alcohol	2-Propanol	67-63-0
	2,2-Dimethyl-1-propanol	75-84-3
	*n*-Hexanol	111-27-3
Ketone	2-Pentanone	107-87-9

2-Propanol was found to be increased in PsA patients compared to controls and compared to RA patients; however, there was no significant difference between RA patients and controls. 2,2-Dimethyl-1-propanol was found to be decreased in RA patients compared to both controls and PsA patients, while it was found to be increased in PsA patients compared to controls. *n*-Hexanol and 2-pentanone were found to be decreased in both RA patients compared to controls and RA patients compared to PsA patients. There was no significant difference in either compound for PsA patients compared to controls. The group did not hypothesize the origin of these VOCs, but said they are most likely a result of the body’s response to the inflammation associated with RA and PsA. Efforts should be made to correlate these reported VOCs to certain pathways and regulatory mechanisms using the quantitative means outlined in the section below. Without these correlations, it is difficult to confidently attribute these reported VOCs to RA or PsA as they may be due to confounding factors.

## Challenges

In order for VOC measurement to be translated to clinical practice, 2 issues must be addressed. First, clear identification of the VOCs and their biochemical origin must be attained. Second, the inter- and intraindividual variation in VOCs found in both diseased and healthy individuals must be better understood. This section outlines these challenges and discusses how they may be addressed.

### Identifying molecular underpinnings of VOC production

A prevalent obstacle preventing VOC biomarkers from being validated biomarkers for clinical use lies in the current knowledge gap regarding the origins of these compounds, specifically the cellular (immune cells, cancer cell, bacterial, etc.) sources and biochemical pathways involved. This information could elucidate the physiological functions of many volatiles and lead to the identification of common VOC biomarkers for certain diseases, such as the ones discussed in this review. Many studies conduct literature searches to identify potential biochemical origins of their identified potential VOC biomarkers, but a handful of studies also present analytical methods of investigating the molecular origins of VOCs. Jia et al. [73] calculated a Pearson’s correlation coefficient between selected VOCs and cellular metabolites and Lima et al. [25] calculated a Spearman’s correlation coefficient between the VOCs they identified to be significantly altered in the urine of PC patients. Correlation is a statistical measure to calculate the association between 2 variables [87]. The coefficient of correlation demonstrates the degree to which changes in one variable are correlated to changes of another variable [87]. The Pearson’s coefficient evaluates the linear relation of variables while the Spearman’s coefficient evaluates the monotonic relation of variables [58]. Jia et al. was able to determine the correlation between multiple VOCs and levels of fatty acids, amino acids, glucose, and cholesterol, which could signal their involvement in certain metabolic pathways. Lima et al. were able to determine a strong correlation between certain VOCs, indicating that they may be in the same metabolic pathway or under a common regulatory mechanism. Furuhashi et al. [72] approached the challenge of investigating the mechanisms behind VOC production in LC and noncancer lung cells by coupling SPME-GC-MS with enzymatic assays in order to determine significant enzymes related to the production of their putatively identified biomarker VOCs of LC. They performed ALDH assays to quantify ALDH activity in order to assess the conversion of aldehyde groups into carboxylic acid groups, phenol sulfuric acid assays to quantify neutral sugars and the speed of their uptake as the main energy source in cells to evaluate the glycolysis pathway, lactate assays to quantify the lactate secreted into the medium as it reflects the balance of reduction and oxidation inside the cells, and reactive oxygen species (ROS) assays to determine if cells were under oxidative stress. These more quantitative means of determining the biochemical pathways and cellular origins of reported potential VOC biomarkers could expedite the use of VOCs as clinical biomarkers of disease.

### The lack of standardization and the impact of confounding factors

**Fig. 5.**
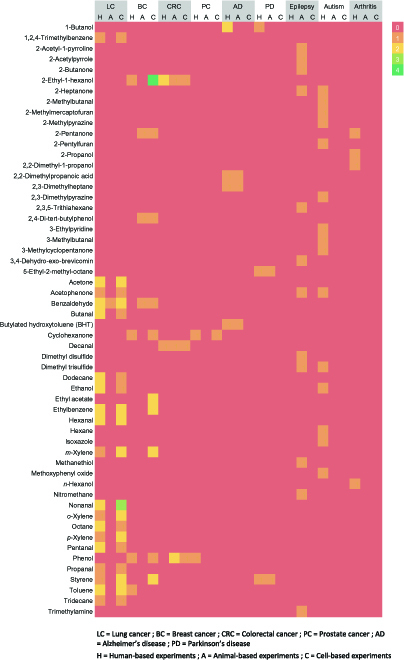
Heat map of all VOCs reported in this review as potential biomarkers of disease. Numbers 0 to 4 correspond to the number of studies reporting that particular VOC.

Despite the seeming success of many studies to identify VOCs with potential diagnostic capabilities, detected and reported volatiles vary notably between study groups and between systems of study. Figure 5 shows a heat map of all the potential VOC biomarkers highlighted in this review, which emphasizes the infrequency of overlapping potential VOC biomarkers reported. An obvious challenge contributing to the diversity in reported volatiles is the variation and lack of standardization in the collection and analysis of samples. The development of standard VOC sampling procedures for a variety of biofluids and the evaluation and quantification of the main sampling factors affecting sample analysis output are formidable challenges for the field, but without this knowledge, VOC analysis will not gain use in clinical practice. Some of the confounding factors found in the studies considered in this review are age [15], gender [22], medication usage [15,31], environmental exposure [19], and time of day of sample acquisition [23]. While in vitro studies have been conducted to avoid the influence of the previously mentioned confounding factors, the inability of conventional cell cultures to model the complex cell–cell and cell–matrix interactions that take place in the body is a limitation of this study system. However, the integration of preliminary in vitro studies for hypothesis generation, studies utilizing animal models for revealing disease-specific metabolic signatures, and human in vivo studies for validation could be key in the transition of VOC biomarkers to clinical practice.

## Conclusions

Advancements in sampling technique in tandem with progress in both GC-MS-based and sensor-based analytical methods have provided a means of understanding the identity of VOCs related to disease and their diagnostic potential, respectively. However, issues concerning sampling techniques, such as a lack of standardization, and analytical methods, such as the expense, sensitivity, and specificity, are obstacles for VOCs as diagnostic tools. A focus on developing consistent sampling procedures and a streamlined process for data analysis for both GC-MS and sensor-based techniques would considerably improve the feasibility of VOCs being used as diagnostic tools for clinical use. Improvements in sampling and analysis would inform elucidation of the mechanisms underlying the production of certain VOCs, which could further inform the role of reported VOCs in the pathogenesis of disease. As the knowledge surrounding the origins of excreted VOCs increases, the devices made to detect and analyze them will become more sophisticated and accessible. For example, the recent coronavirus disease 2019 (COVID-19) pandemic has prompted further advancements in VOC detection, specifically in the exhaled breath due to the noninvasive nature of its collection procedure. The company Worlds Inc. partnered with Texas A&M as well as the U.S. Air Force to test their prototypes of Worlds Protect, a kiosk that can be placed outside to rapidly test an individual’s exhaled breath for COVID-19 through the use of MS with artificial intelligence applied to improve the accuracy of results as more samples are tested [88]. These quick, accessible, and easily understood kiosks that do not require expert handling have the potential to greatly influence public health. Places of high traffic, such as airports, restaurants, and hotels, could implement these kiosks for the safety of their customers in the event of another global pandemic or even during flu season. An integral aspect of the development of Worlds Protect was the availability of PCR testing as a gold standard for comparison in order to understand the progress being made in their VOC study [88]. While these diagnostic gold standards have high sensitivity for infectious diseases, such as COVID-19, finding these standards for comparison for noninfectious diseases, such as those discussed in this review, is more difficult as early diagnosis is the key aspect that VOC biomarkers would support. Owlstone Medical is currently conducting a multicenter clinical trial for the diagnosis of LC based on the concentration of VOCs in the exhaled breath [74]. Since there is no gold standard test for the early detection of LC, eligibility is based on the suspicion of LC and the study must follow the patients while their diagnosis is confirmed. The future of early diagnosis for noninfectious diseases depends on the advancements in diagnostic tools and standardized procedures. All these improvements together will ultimately enable alternatives for diagnosing and treating disease in a more accessible manner.
